# Proteomic Analysis and Functional Characterization of P4-ATPase Phospholipid Flippases from Murine Tissues

**DOI:** 10.1038/s41598-018-29108-z

**Published:** 2018-07-17

**Authors:** Jiao Wang, Laurie L. Molday, Theresa Hii, Jonathan A. Coleman, Tieqiao Wen, Jens P. Andersen, Robert S. Molday

**Affiliations:** 1 Department of Biochemistry and Molecular Biology, Centre for Macular Research, University of British Columbia, Vancouver, British Columbia V6T 1Z3 Canada; 2 Laboratory of Molecular Neural Biology, Institute of Systems Biology, School of Life Sciences, Shanghai University, Shanghai, 200444 China; 3 Department of Biomedicine, Aarhus University, Ole Worms Allé 4, Bldg. 1160, DK-8000, Aarhus C, Denmark

## Abstract

P4-ATPases are a subfamily of P-type ATPases that flip phospholipids across membranes to generate lipid asymmetry, a property vital to many cellular processes. Mutations in several P4-ATPases have been linked to severe neurodegenerative and metabolic disorders. Most P4-ATPases associate with one of three accessory subunit isoforms known as CDC50A (TMEM30A), CDC50B (TMEM30B), and CDC50C (TMEM30C). To identify P4-ATPases that associate with CDC50A, *in vivo*, and determine their tissue distribution, we isolated P4-ATPases-CDC50A complexes from retina, brain, liver, testes, and kidney on a CDC50A immunoaffinity column and identified and quantified P4-ATPases from their tryptic peptides by mass spectrometry. Of the 12 P4-ATPase that associate with CDC50 subunits, 10 P4-ATPases were detected. Four P4-ATPases (ATP8A1, ATP11A, ATP11B, ATP11C) were present in all five tissues. ATP10D was found in low amounts in liver, brain, testes, and kidney, and ATP8A2 was present in significant amounts in retina, brain, and testes. ATP8B1 was detected only in liver, ATP8B3 and ATP10A only in testes, and ATP8B2 primarily in brain. We also show that ATP11A, ATP11B and ATP11C, like ATP8A1 and ATP8A2, selectively flip phosphatidylserine and phosphatidylethanolamine across membranes. These studies provide new insight into the tissue distribution, relative abundance, subunit interactions and substrate specificity of P4-ATPase-CDC50A complexes.

## Introduction

P4-ATPases comprise a subfamily of P-type ATPases that actively flip phospholipids from the exocytoplasmic to the cytoplasmic leaflet of cell membranes. This unidirectional transport generates phospholipid asymmetry across cell membranes, a property that has been linked to a variety of crucial cellular processes including cell and organelle shape determination, cell division, vesicle budding and transport, fertilization, phagocytosis, apoptosis, regulation of membrane protein function, blood coagulation, axonal elongation, and sensory function among others^1–4^.

The importance of P4-ATPases is highlighted by the finding that a number of these phospholipid transporters have been linked to severe genetic diseases. In mice, loss of function mutations in P4-ATPases cause neurodegenerative diseases, sensory defects, cholestasis, obesity, and blood and immune abnormalities^5–12^. In humans, mutations in specific P4-ATPases have been linked to progressive familial intrahepatic cholestasis^13^, severe neurological and motor disorders^14,15^, and congenital hemolytic anemia^16^.

Like other P-type ATPases, P4-ATPases contain a large catalytic or α-subunit composed of a nucleotide binding domain (N-domain), a phosphorylation domain (P-domain), and an actuator domain (A-domain) which are involved in the ATP reaction cycle, and a membrane domain (M-domain) consisting of 10 predicted transmembrane segments which serve as the pathway for the translocation of phospholipids across cell membranes^1,17–21^. In addition, some mammalian P4-ATPases contain an extended C-terminal segment implicated in protein folding and regulation of its activity^22^. The human genome encodes 14 P4-ATPases which have been organized into 5 subclasses based on the sequence similarity of their catalytic subunits: Class 1a (ATP8A1, ATP8A2); Class 1b (ATP8B1, ATP8B2, ATP8B3, ATP8B4); Class 2 (ATP9A, ATP9B); Class 5 (ATP10A, ATP10B, and ATP10D); and Class 6 (ATP11A, ATP11B, ATP11C).

With the exception of ATP9A and ATP9B, the P4-ATPase catalytic subunits associate with an evolutionary conserved accessory or β-subunit known as either CDC50 or TMEM30 to form a heteromeric complex^12,23–27^. CDC50 proteins consist of two transmembrane segments joined by a highly glycosylated exocytoplasmic segment. Interaction of CDC50 with the α-subunit is essential for the proper folding, transport activity, and exit of the P4-ATPase complexes from the endoplasmic reticulum^12,25,26,28–31^. In mammals, there are 3 isoforms: CDC50A; CDC50B; and CDC50C.

The interaction of P4-ATPases with CDC50 variants has been primarily studied using heterologous co-expression and co-immunoprecipitation^12,24–26,32^. In this experimental system, ATP8A1, ATP8A2, ATP11A-C, ATP10A, ATP10B, and ATP10D were found to selectively assemble with CDC50A, whereas several members of Class 1b including ATP8B1 and ATP8B2 were found to associate with CDC50B as well as CDC50A. The P4-ATPase that interacts with CDC50C has not been identified. Although CDC50 subunits play a crucial role in the proper folding and functional activity of P4-ATPases, the CDC50 variants that associate with specific P4-ATPases in native tissues have not been identified, and the effect of the CDC 50A and CDC50B variants on the functional properties of Class1b P4-ATPase remains to be investigated.

The transport activities of several mammalian P4-ATPase-CDC50A complexes have been determined. ATP8A2 and ATP8A1 complexes purified and reconstituted into liposomes have been shown to actively transport phosphatidylserine (PS) and to a lesser degree phosphatidylethanolamine (PE) across the lipid bilayer^25,33,34^. ATP11C and ATP11A have also been implicated as PS and PE flippases using cell based assays, but the substrate for ATP11B is not known^35^. ATP8B1 was originally suggested to transport PS^29^, but more recently ATP8B1, ATP8B2, and ATP10A, have been reported to selectively transport PC using cell-based assays^32,35^. ATP8B3 has been implicated in the transport PS based on its role in maintaining PS asymmetry and fertilization in spermatozoa in mice^36^, but direct transport assays have not been reported. The substrates of other P4-ATPases including ATP11B, ATP8B4, ATP10B, ATP10D, ATP9A and ATP9B remain to be determined.

Although considerable progress has been made on the molecular characterization of many P4-ATPases by heterologous cell expression, to date there is only limited information on the distribution, abundance, and molecular properties including subunit interactions of specific P4-ATPases in native cells and tissues^27,33,37,38^. This is due in part to the low level of expression of many P4-ATPases and the paucity of highly specific and sensitive antibodies for immunolocalization and immunoprecipitation of specific flippases from various tissues and cells.

In this study we have developed a highly sensitive immunoaffinity-based mass spectrometric method to identify high and low abundant P4-ATPase-CDC50A complexes in five mouse tissues including retina, testes, liver, brain, and kidney. Ten distinct complexes were detected which varied in tissue distribution and abundance. We have also expressed and purified ATP11-CDC50A complexes for analysis of their phospholipid dependent ATPase and flippase activities. We show that ATP11B as well as ATP11A and ATP11C specifically transport PS and PE across cell membranes and define their kinetic parameters.

## Results

### Proteomic analysis and distribution of P4-ATPase-CDC50A complexes in neural retina

In previous studies, we showed that retinal photoreceptors and ganglion cells express ATP8A2-CDC50A complexes^6,33^. To determine if the CDC50A antibody Cdc50-7F4 can be used to co-immunoprecipitate ATP8A2 and other P4-ATPases that associate with CDC50A, we applied a detergent-solubilized retinal extract to an immunoaffinity column consisting of a Cdc50-7F4 monoclonal antibody covalently coupled to Sepharose 2B. After removal of the unbound proteins, the P4-ATPase-CDC50A complexes were eluted from the affinity matrix with the competing 7F4 peptide. The eluent was subjected to in-gel trypsin digestion and the resulting peptides were analyzed by tandem mass spectrometry (MS/MS). Five P4-ATPases (ATP8A2, ATP8A1, ATP11A, ATP11B, and ATP11C) along with CDC50A were identified with a high degree of confidence (Table 1). In each of three independent experiments, the highest number of peptides was derived from ATP8A2 and ATP8A1 with a lower number from ATP11B, ATP11A and ATP11C. Spectral intensity measurements also indicated that ATP8A2 and ATP8A1 were present at significantly higher abundance than ATP11A, ATP11B, and ATP11C. In control experiments, neither P4-ATPases nor CDC50A were detected when a retina extract was applied to a column containing an immobilized control antibody (see Supplemental Table S6).

**Table 1:** Mass Spectrometric Analysis of P4-ATPases in Mouse Tissues.

Tissue	Protein ID	Protein Name	Mwt (daltons)	Peptides	% Sequence Coverage	% Intensity
Retina	Q8VEK0	CDC50A/TMEM50A	41,061	7	22.5	—
	P98200	ATP8A2	129,417	31	32.1	45.3
	P70704	ATP8A1	131,413	29	32.3	36.5
	Q6DFW5	ATP11B	133,536	21	23.7	12.4
	Q9QZW0	ATP11C	129,240	10	12.2	2.9
	P98197	ATP11A	135,502	14	15.1	2.9
Liver	Q8VEK0	CDC50A/TMEM50A	41,061	24	61.9	—
	Q9QZW0	ATP11C	129,240	114	60.7	95.20
	Q148W0	ATP8B1	143,798	29	31.2	1.01
	P98197	ATP11A	135,502	26	33.8	0.97
	P70704	ATP8A1	131,413	39	41.9	2.54
	Q6DFW5	ATP11B	133,536	35	38.0	0.09
	Q8K2X1	ATP10D	158,330	10	12.1	0.09
	P98200	ATP8A2	129,417	4	4.2	0.06
Brain	Q8VEK0	CDC50A/TMEM50A	41,061	27	49.7	—
	P70704	ATP8A1	131,413	77	51.2	56.53
	Q9QZW0	ATP11C	129,240	46	38.2	12.85
	P98200	ATP8A2	129,417	43	34.7	13.20
	Q6DFW5	ATP11B	133,536	42	34.9	8.56
	P98197	ATP11A	135,502	36	33.5	8.33
	P98199	ATP8B2	136,968	14	18.2	0.27
	Q8K2X1	ATP10D	158,330	14	13.9	0.23
Testes	Q8VEK0	CDC50A/TMEM50A	41,061	19	49.7	—
	Q6UQ17	ATP8B3	151,955	55	42.2	18.08
	Q9QZW0	ATP11C	129,240	53	40.3	23.12
	Q6DFW5	ATP11B	133,536	48	43.5	23.97
	P70704	ATP8A1	131,413	38	39.6	7.03
	P98197	ATP11A	135,502	37	33.8	20.41
	P98200	ATP8A2	129,417	37	27.5	5.18
	Q8K2X1	ATP10D	158,330	21	18.0	1.25
	O54827	ATP10A	168,788	17	15.1	0.93
	P98199	ATP8B2	136,968	4	4.6	0.16
Kidney	Q8VEK0	CDC50A/TMEM50A	41,061	23	47.8	—
	P98197	ATP11A	135,502	58	37.4	81.12
	Q9QZW0	ATP11C	129,240	45	36.7	13.64
	P70704	ATP8A1	131,413	32	31.6	2.10
	Q6DFW5	ATP11B	133,536	28	25.7	2.99
	Q8K2X1	ATP10D	158,330	8	7.5	0.11

A detergent solubilized mouse tissue extract (~20 mg) was applied to a Cdc50-7F4 immunoaffinity column. After removing the unbound protein, the P4-ATPase-CDC50A complexes were eluted with the 7F4 competing peptide. The eluted fraction was subjected to in-gel (retina) or FASP (other tissues) trypsin digestion for analysis of peptides by tandem MS/MS. %Intensity was obtained by taking the fraction of the spectral intensity of a P4-ATPase to the total spectral intensity of all P4-ATPases detected in the tissue.

**Figure 1:**
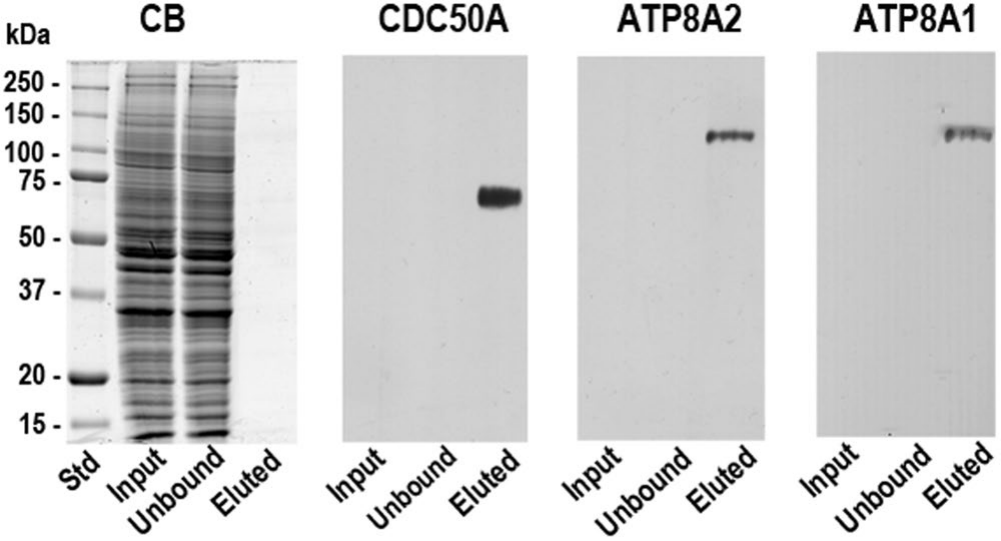
Co-immunoprecipitation of ATP8A2 and ATP8A1 with CDC50A from mouse retina. A retinal extract from mouse retina tissue solubilized in CHAPS detergent was incubated with the immunoaffinity matrix consisting of the Cdc50-7F4 monoclonal antibody covalently coupled to Sepharose 2B. After removal of the unbound fraction, the matrix was washed and eluted with the 7F4 peptide for analysis on SDS gels stained with Coomassie Blue (CB) and western blots labeled with antibodies to CDC50A, ATP8A2 and ATP8A1. The input and unbound lanes contained about 30 µg of protein. Although the P4-ATPases were not detected by Coomassie blue staining due to their low abundance, the ATP8A2, ATP8A1 and CDC50A could be readily detected in the eluted fraction by western blotting.

To validate the immunoaffinity-based detection of P4-ATPase-CDC50A complexes by MS/MS, western blots of the retinal extract, unbound, and peptide eluted fractions were labeled with antibodies to ATP8A2, ATP8A1 and CDC50A. Although the P4-ATPases and CDC50A were not detected in the retinal extract due to the low levels of these proteins, ATP8A2, ATP8A1, and CDC50A were detected in the concentrated peptide-eluted fractions by western blotting (Fig. 1) confirming the results obtained by MS/MS.

**Figure 2:**
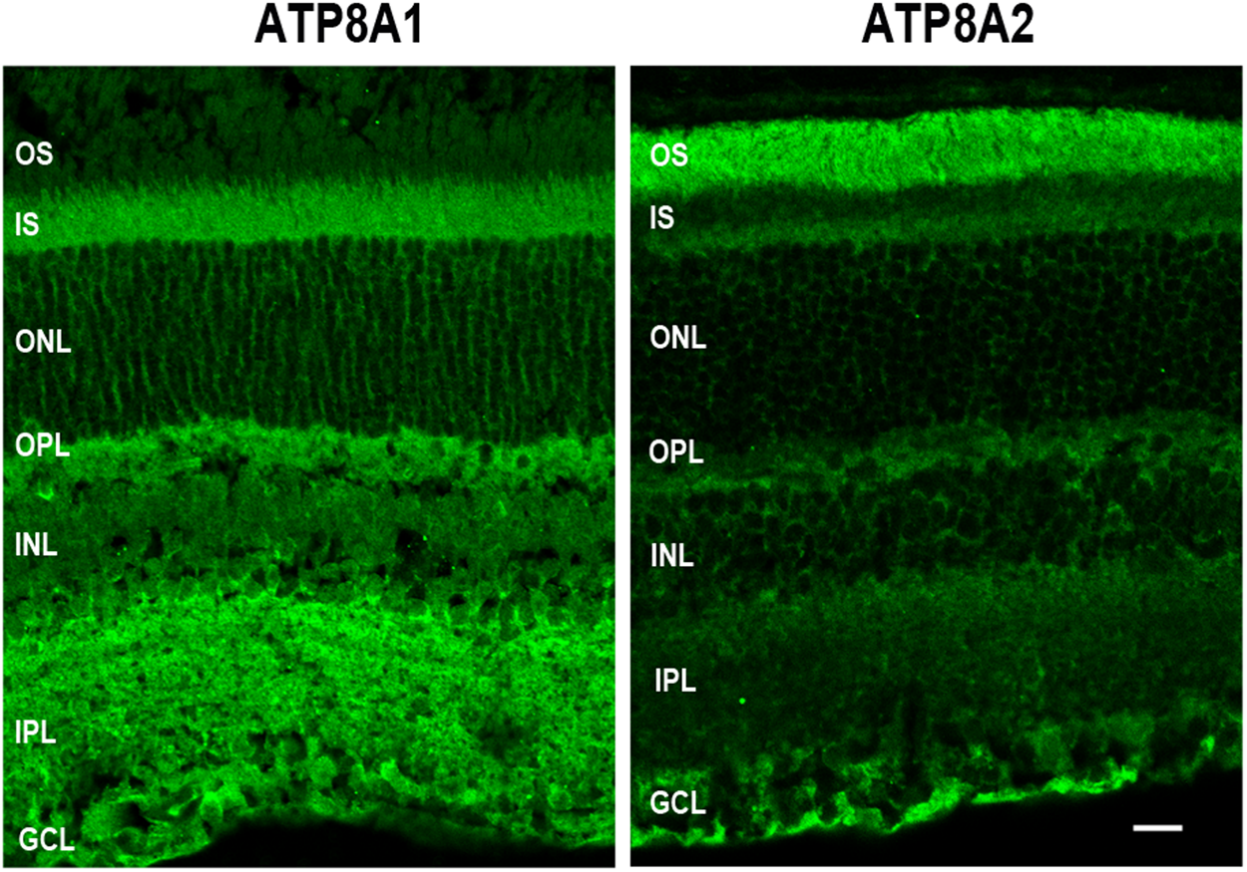
Immunofluorescence localization of ATP8A1 and ATP8A2 in the mouse retina. Cryosections of adult mouse retina were labeled with ATP8A1 and ATP8A2 antibodies for analysis by confocal scanning microscopy. ATP8A1 and ATP8A2 exhibit some differences in cellular and subcellular localization. ATP8A2 is predominantly present in photoreceptor outer segments and ganglion cell layers, but also present in at a lower level in the inner segments, outer plexiform layer and inner retina. ATP8A1 is absent from outer segments but otherwise displays a more universal distribution throughout the retina. OS:outer segment; IS:inner segment; ONL:outer nuclear layer; OPL:outer plexiform layer; INL:inner nuclear layer; IPL:inner plexiform layer; GCL:ganglion cell layer. Bar = 20 µm

The distribution of ATP8A1 in adult mouse retinal cryosections was compared with that of ATP8A2 by confocal scanning microscopy. As shown in Fig. 2, ATP8A1 intensely labeled numerous layers of the retina including the inner segment, outer and inner plexiform layers, inner nuclear layer and the ganglion cells. However, no labeling was observed in the outer segment layer of photoreceptor cells. In contrast, intense ATP8A2 labeling was seen in the outer segments and ganglion cell layer with weaker labeling in the inner segment layer and the outer and inner plexiform layers (Fig. 2). Efforts to label ATP11A and ATP11C on western blots and retinal cryosections were unsuccessful most likely due to the low abundance of these P4-ATPases in the retina (Table 1) and/or the lower sensitivity of these antibodies.

### Detection of P4-ATPase-CDC50A complexes in murine tissues by western blotting

**Figure 3:**
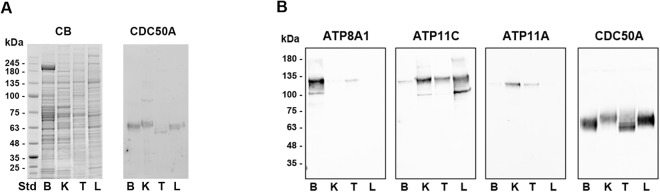
SDS gels and Western blots of P4-ATPase complexes from brain, kidney, testes, and liver. (**A**) Proteins from membrane fractions of mouse brain (B), kidney (K), testes (T), and liver (L) were resolved on a SDS gel and either stained with Coomassie Blue (CB) or transferred to Immobilon membranes and labeled for CDC50A with the Cdc50-7F4 monoclonal antibody. Approximately, 30 µg of protein was applied to each lane. (**B**) Western blots of P4-ATPases in mouse tissues isolated by immunoaffinity chromatography. Membranes from brain (B), kidney (K), testes (T), and liver (L) were solubilized in CHAPS detergent and P4-ATPase complexes were isolated by immunoaffinity chromatography on a Cdc50-7F4 immunoaffinity matrix. Western blots were labeled with antibodies to ATP8A1, ATP11C, ATP11A and CDC50A.

To determine if CDC50A can be detected in other tissues, we resolved proteins from membranes of brain, kidney, testes, and liver by SDS gel electrophoresis for immunolabeling of western blots (Fig. 3A). CDC50A was detected in all four tissues, migrating with an apparent molecular weight of 60–70 kDa. Variation in the migration of CDC50A is most likely due to differential glycosylation in the various tissues.

Several P4-ATPases isolated on a CDC50A immunoaffinity matrix from detergent-solubilized brain, kidney, testes, and liver membranes were analyzed by Western blotting (Fig. 3B). ATP11C was detected in all four tissues with liver displaying the highest level of expression and brain the lowest level. ATP11A was abundant in kidney and more faintly detectable in testes and brain, whereas ATP8A1 was most abundant in brain and detectable in testes. Western blot validated antibodies to other P4-ATPases were not generally available as part of this study.

### Mass-spectrometry-based proteomic analyses of P4-ATPase-CDC50A complexes in other mouse tissues

Affinity-based mass spectrometry was used to identify P4-ATPase-CDC50A complexes and determine their relative amounts in various tissues. Proteins isolated from liver, brain, testes and kidney extracts on a CDC50A immunoaffinity matrix were subjected to trypsin digestion for analysis of the peptides by MS/MS. As part of this study, we compared the peptides from liver-derived P4-ATPase-CDC50A complexes obtained by in-gel trypsin digestion with those obtained from the filter-aided sample preparation (FASP) procedure^39^. Both methods generated tryptic fragments that identified the same P4-ATPases, although the FASP procedure produced several fold more peptides than in-gel digestion (see Supplemental Table S3).

Tryptic peptides prepared by the FASP procedure identified CDC50A in all tissues by MS/MS with sequence coverage ranging from 48% to 62% and multiple P4-ATPases (Table 1). On the basis of spectral intensity measurements, the most abundant P4-ATPases were ATP11C (95% of total P4-ATPases) in liver, ATP8A1 (57%) in brain, ATP11A (81%) in kidney, and various P4-ATPases including ATP8B3 (18%), ATP11C (23%), ATP11B (24%), and ATP11A (20.4%) in testes. ATP11A, ATP11B, ATP11C, ATP8A1 and ATP10D were found in all four tissues, whereas ATP8A2 was found in significant amounts in brain and testes. In liver ATP8B1 was also present indicating that a portion of this P4-ATPase associates with CDC50A *in vivo* in agreement with heterologous cell expression studies^24,29^.

### Other proteins that co-immunoprecipitate with P4-ATPase-CDC50A complexes

In addition to P4-ATPase-CDC50A complexes, other proteins were detected in significant levels in the eluted fraction from the CDC50A immunoaffinity column. Examples of proteins found in significant quantities in the eluted fraction from all or most tissues included clathrin, a major protein involved in the formation of coated vesicles and associated with vesicle trafficking, catenins, a family of proteins which are known to interact with the cell adhesion proteins cadherins, endoplasmic reticulum chaperone BiP encoded by the Hspa5 gene, and various metabolic enzymes. Listings of the most abundant proteins are given in the Supplemental Tables (Tables S1-S5). Whether these proteins and other proteins in the data sets are true interacting proteins or residual contaminants require further studies.

### Expression, purification and ATPase activity of ATP11-CDC50A complexes

**Figure 4:**
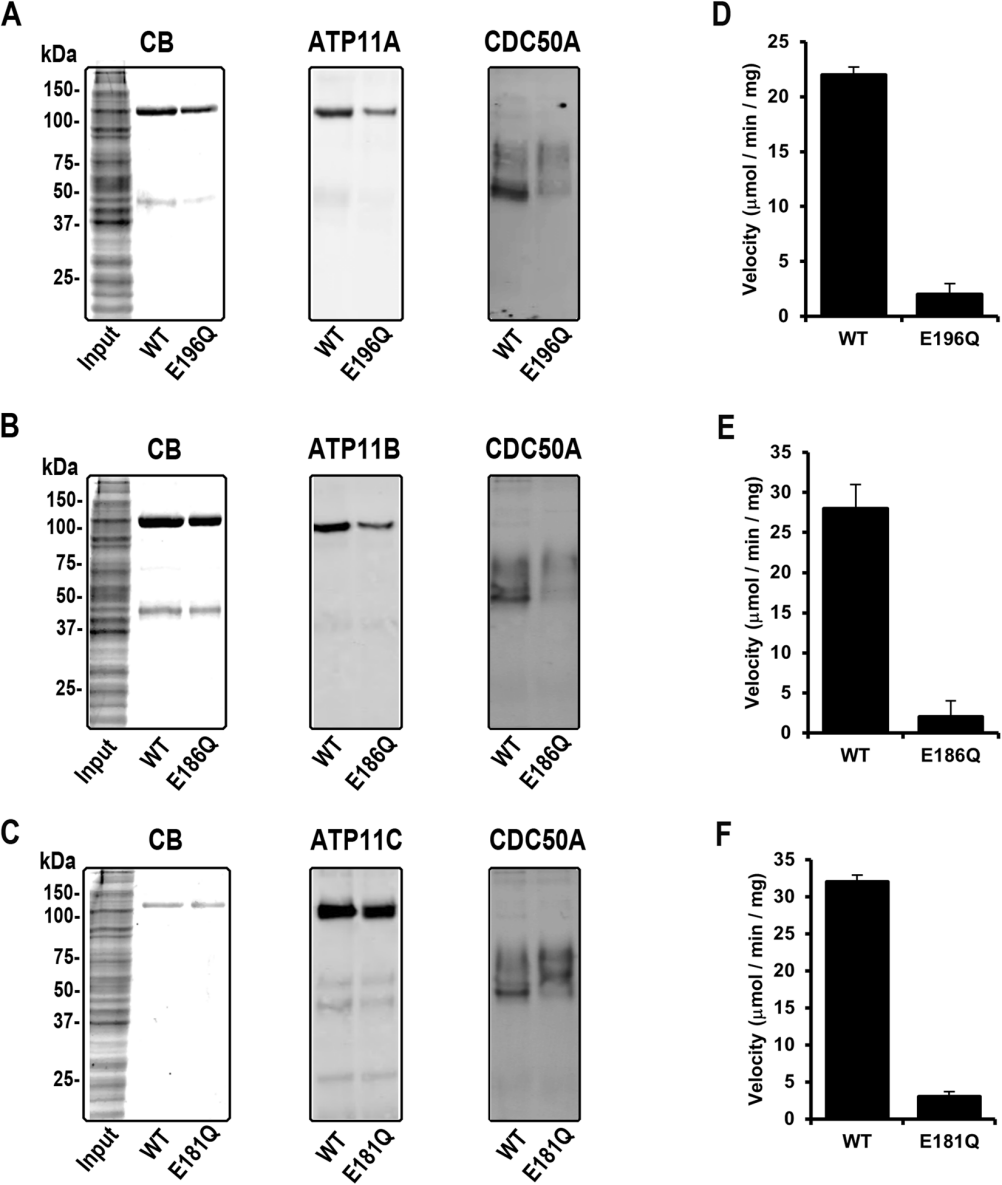
Purification and ATPase activity of WT ATP11 and mutants with the E→Q mutation in the activator domain. ATP11A, ATP11B, and ATP11C containing a 1D4 tag were co-expressed with CDC50A in HEK293 cells and purified by immunoaffinity chromatography on a Rho1D4-Sepharose matrix. SDS gels and Western blots of the HEK293 cell extracts (Input) and 1D4 peptide eluted WT or E→Q mutants for ATP11A (**A**), ATP11B (**B**) and ATP11C (**C**). SDS gels were stained with Coomassie Blue (CB) and Western blots were labeled with the Rho 1D4 antibody (ATP11) or Cdc50-7F4 antibody (CDC50A). ATPase activity for WT and E→Q mutants in the presence of brain polar lipid is shown for ATP11A (**D**), ATP11B (**E**), and ATP11C (**F**). Data shown as the mean ± SD for n = 3.

As shown above, ATP11A, ATP11B, and ATP11C are widely expressed in mouse tissues. To begin to determine their molecular properties and function as phospholipid flippases, we co-expressed CDC50A in HEK293T cells together with 1D4-C-terminal tagged ATP11A, ATP11B and ATP11C and their corresponding ATPase-deficient mutants in which glutamate in the DGET motif of the A-domain was replaced with glutamine [E→Q]. The expressed ATP11-CDC50A complexes were purified on a Rho 1D4 immunoaffinity matrix for analysis of their functional properties. As shown in Fig. 4A–C, the purified WT and mutant ATP11 proteins migrated on SDS gels as a major Coomassie blue stained band having an apparent molecular weight in the range of 120 to 130 kDa. The highly glycosylated CDC50A which co-purifies with the WT and mutant ATP11 proteins ran as a relatively broad band as observed by western blotting. A 45 kDa protein was also detected on Coomassie blue stained gels of purified WT and mutant ATP11A and ATP11B. This protein may represent a proteolytic fragment of the ATP11 proteins or another associated protein.

The ATPase activity of the purified WT ATP11-CDC50A complexes and their E→Q mutants was first determined in the presence of brain polar lipid (BPL) (Fig. 4D–F). The WT ATP11A, ATP11B, and ATP11C complexes displayed significant ATPase activity whereas the E→Q mutants showed only background levels consistent with the absence of contaminating ATPases in these preparations and the essential role of the glutamate residue in the DGET motif of P-type-ATPases as previously reported^18,40^.

### Activation of ATPase activity of ATP11-CDC50 complexes by specific phospholipids

**Figure 5:**
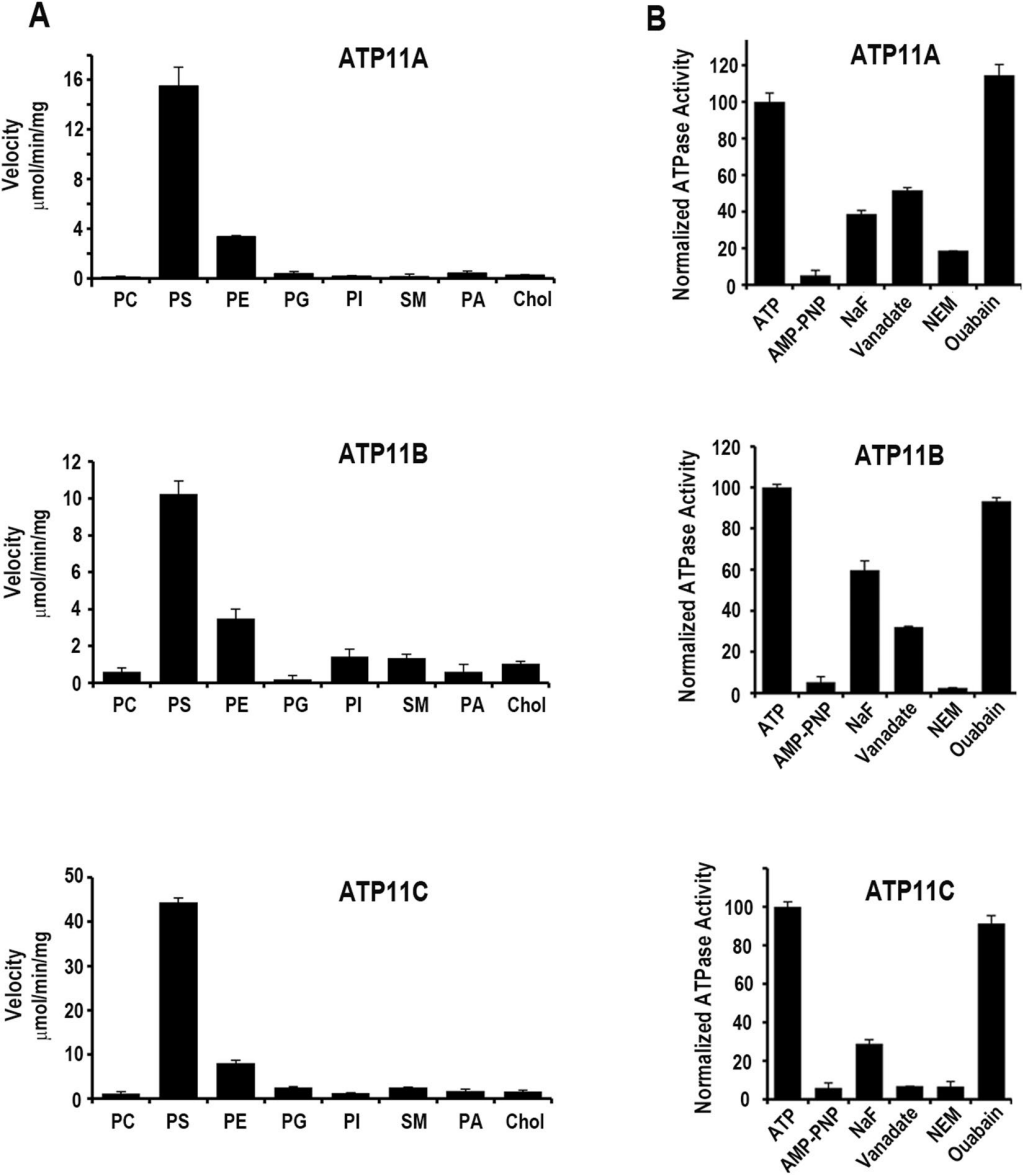
Effect of specific phospholipids, nucleotides and inhibitors on the ATPase activity of ATP11 proteins. (**A**) ATP11A, ATP11B and ATP11C were co-expressed with CDC50A in HEK293 cells, purified by immunoaffinity chromatography, and reconstituted with 100% DOPC (PC) or 90% DOPC and 10% DOPS (PS), DOPE (PE), DOPG (PG), DOPI (PI), sphingomyelin (SM), DOPA (PA) or cholesterol (Chol) for determination of their ATPase activity. (**B**) The ATPase activities were determined for 0.5 mM ATP or 0.5 mM non-hydrolyzable ATP analogue AMP-PNP. For inhibition studies, the proteoliposomes were pre-incubated with 100 μM NaF, 100 μM vanadate, 5 mM N-ethylmaleimide (NEM), or 1 mM ouabain prior to the addition of 0.5 mM ATP for ATPase measurements. The ATPase activity was normalized to the activity of proteoliposomes in the presence of ATP, but in the absence of inhibitors. Data shown as the mean ± SD for n = 3.

The effect of specific membrane lipids on the ATPase activity of ATP11A, ATP11B and ATP11C complexes was studied by comparing the rate of ATP hydrolysis in 90% DOPC and 10% specific lipid with the rate in 100% DOPC. Figure 5A shows that the ATPase activities of ATP11A, ATP11B, and ATP11C in 100% DOPC was negligible whereas the ATPase activity of these P4-ATPases was significantly activated by dioleylphosphatidylserine (DOPS) and to a lesser extent dioleylphosphatidylethanolamine (DOPE). In contrast, little if any ATPase activity was observed for cholesterol, sphingomyelin (SM) or the dioleylphospholipids, phosphatidylglycerol (DOPG), phosphatidylinositol (DOPI), or phosphatidic acid (DOPA). The specific ATPase activity of ATP11C in the presence of PS or PE was generally higher than that for ATP11A or ATP11B (Table 2).

**Table 2:** Kinetic Parameters for ATP11-CDC50A Complexes.

Substrate	ATP11A	ATP11B	ATP11C	ATP8A2^‡^	ATP8A1^§^
PS					
K_A_ (µM)	87 ± 23	178 ± 19	69 ± 13	38 ± 1	39 ± 4
Vmax	15.1 ± 1.1	17.3 ± 0.6	34.9 ± 1.7	160 ± 6^#^	38.7 ± 1
PE					
K_A_ (µM)	240 ± 40	5470 ± 1200	2650 ± 1120	371 ± 13	887 ± 111
Vmax	12.2 ± 0.6	25.2 ± 4.4	48.4 ± 13.7	67 ± 3	26.8 ± 1.5
ATP					
Km (µM)	200 ± 50	730 ± 190	1440 ± 110	704 ± 7	267 ± 5
Vmax	9.6 ± 0.4	9.5 ± 0.7	48.3 ± 1.5	57 ± 2	37.6 ± 2

Data is the mean ± SEM. Vmax is in µmoles ATP hydrolyzed per min per mg protein.

^‡^Data from ref.^19,33^.

^#^Vmax for PS activation of ATP8A2 varies from 80–160 µmoles ATPase hydrolyzed per min per mg protein.

^§^Data from ref.^34^.

The effect of various nucleotides and inhibitors on the ATPase activity of ATP11A, ATP11B and ATP11C was also investigated. As shown in Fig. 5B, no significant PS-activated ATPase activity was observed when the nonhydrolyzable derivative AMP-PNP was used in place of ATP or when the sulfhydryl modifying reagent N-ethylmaleimide (NEM) was added as an inhibitor. NaF and vanadate inhibitors showed a partial reduction in PS-activated ATPase activity, whereas ouabain had no effect.

**Figure 6:**
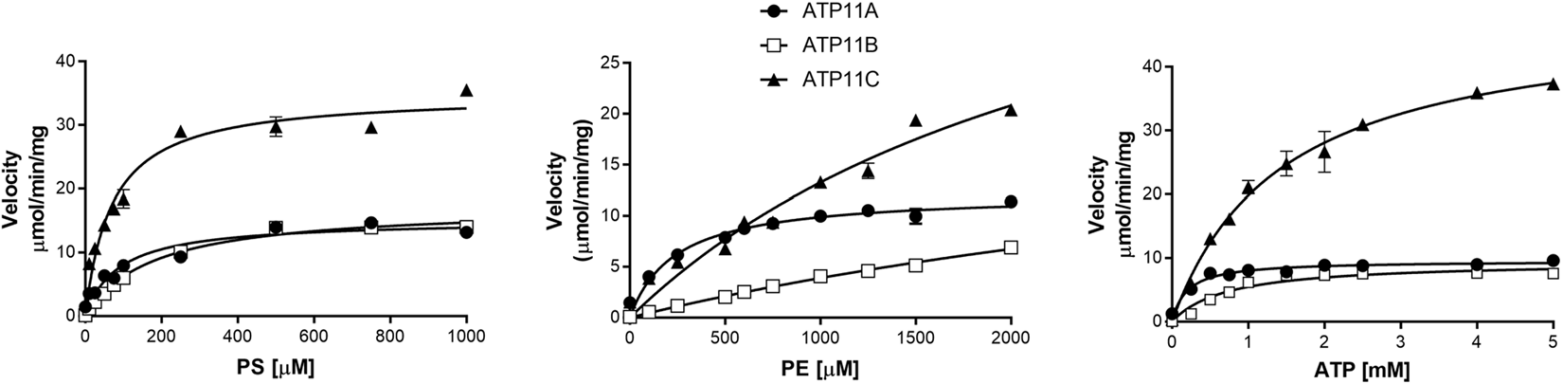
The effect of PS, PE and ATP concentration on the ATPase activity of purified ATP11A-CDC50A, ATP11B-CDC50A, and ATP11C- CDC50A complexes isolated from transfected HEK293 cells. The purified proteins were reconstituted with DOPC as the base phospholipid and varying concentrations of DOPS **(A**) or DOPE (**B**) and the ATPase assays were carried out with 5 mM ATP. (**C**) The effect of ATP concentration on the ATPase activity for ATP11A, ATP11B and ATP11C complexes reconstituted with 70% DOPC and 30% DOPS. Measurements were performed in triplicate and results were averaged. Error bars represent ± SD. Curves were fitted with a Michaelis-Menten equation using the parameters summarized in Table 2.

The effect of DOPS concentration on the ATPase activity of ATP11-CDC50A complexes is shown in Fig. 6A. All three ATP11 variants exhibited saturation curves with a half-maximum activation (K_A_) values ranging from 70 to 178 μM and V_*max*_ values from 15 to 35 μmol ATP hydrolyzed/min/mg protein for DOPS (Table 2). The effect of DOPE concentrations on the ATPase activities of the ATP11 proteins was also determined (Fig. 6B). The apparent K_A_ values for DOPE were significantly higher than for DOPS, whereas the apparent V_*max*_ values were generally similar to values for DOPS (Table 2).

The rate of ATP hydrolysis by ATP11 complexes as a function of ATP concentration was also compared (Fig. 6C). Michaelis-Menten kinetics were observed for all three P4-ATPase-CDC50A complexes with Km values ranging from 0.20 to 1.44 mM and V_*max*_ values from 9.5 to 48 μmol ATP hydrolyzed/min/mg protein in the presence of DOPS (Table 2).

### Phospholipid flippase activities of ATP11-CDC50A complexes

Although phospholipid activation of the ATPase activity can provide some insight into plausible substrates for ATP11 proteins, direct measurement of phospholipid transport or flipping by P4-ATPases across membranes is essential for further confirming their role as phospholipid specific flippases. ATP-dependent flipping of fluorescent NBD-labeled phospholipids can be used to directly examine flippase activity of P4-ATPases in liposomes^33^. We first examined the feasibility of using this assay for the ATP11-CDC50A complexes by determining if NBD-labeled PS can activate the ATPase activity of ATP11A, ATP11B and ATP11C. As shown in Fig. 6A, NBD-labeled PS and DOPS both activated the ATPase activity of the ATP11-CDC50A complexes, although activation by NBD-labeled PS was only 20% that of the unlabeled PS.

**Figure 7:**
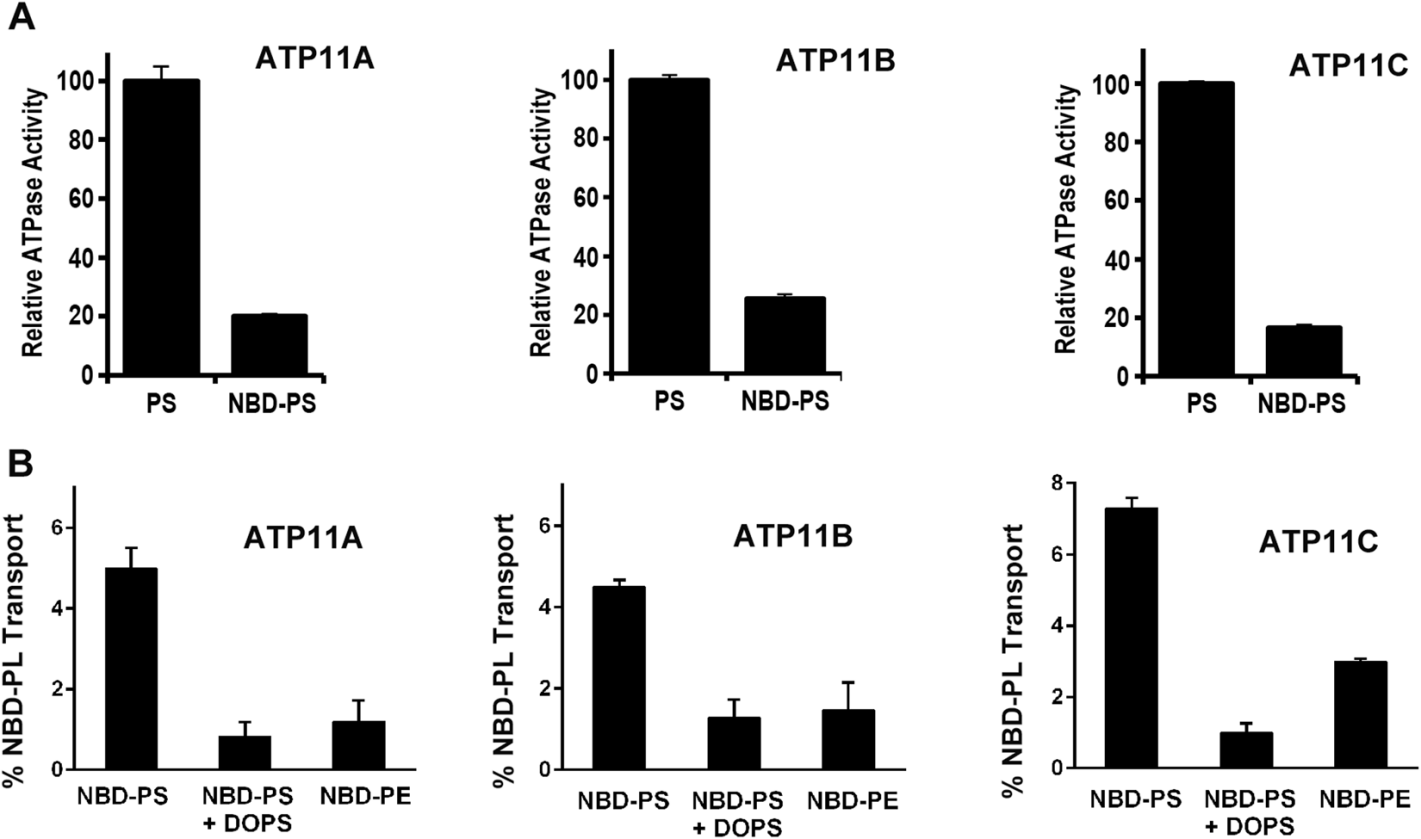
ATPase activity and phospholipid flippase activity of ATP11-CDC50A complexes. (**A**) ATPase activity of ATP11A, ATP11B and ATP11C in the presence of 90% DOPC and either 10% DOPS (PS) or 10% NBD-PS. Activity is normalized to 100% ATPase activity in 10% DOPS. (**B**) NBD-phospholipid flippase activity. ATP11A, ATP11B and ATP11C associated with CDC50A were purified and reconstituted into DOPC liposomes containing 2.5% NBD-PS, 2.5% NBD-PE or 2.5% NBD-PS plus 30% DOPS. The activity was normalized to samples containing NBD-PS. Addition of 30% unlabeled DOPS effectively competed with NBD-PS to reduce the NBD-PS flipping. Data is the average of 3 experiments ± SD.

For flippase assays, ATP11-CDC50A complexes were reconstituted into liposomes consisting of 97.5% egg PC and 2.5% NBD-labeled PS or NBD-labeled PE. The flippase reaction was initiated by the addition of 1 mM ATP or 1 mM AMP-PNP as a control. After 2.5 minutes the reaction was stopped by the addition of buffer containing excess EDTA. Subsequently, dithionite was added to bleach the fluorescent NBD-labeled lipid exposed on the outer leaflet of the vesicles (equivalent to the cytoplasmic side of cell membranes). The decrease in fluorescence between the ATP treated and AMP-PNP-treated samples was used to determine the extent of ATP-dependent NBD-labeled phospholipid transport (Supplemental Fig. S-1 and S-2). As shown in Fig. 7, ATP11A, ATP11B and ATP11C flipped NBD-labeled PS and to a lesser extent NBD-labeled PE. NBD-labeled PS transport was competitively inhibited by adding unlabeled DOPS to the liposomes.

## Discussion

In this study, we have developed a highly sensitive, generally applicable mass spectrometry approach to identify high and low abundance P4-ATPases that use CDC50A as their β-subunit. Ten P4-ATPases were detected in various tissues including ATP8A1, ATP8A2, ATP11A, ATP11B, ATP11C, ATP10A, ATP10D, ATP8B1, ATP8B2 and ATP8B3 (Table 1). Four P4-ATPases (ATP8A1, ATP11A, ATP11B, and ATP11C) were present in all five tissues examined. ATP10D was found in low amounts in liver, brain, testes, and kidney, and ATP8A2 was present in significant amounts in retina, brain, and testes in agreement with gene expression profiles and western blotting^33,41^. Several P4-ATPases had a more restricted tissue distribution. ATP8B1 was detected only in liver, ATP8B2 primarily in brain, and ATP8B3 and ATP10A in testes. Interestingly, each tissue differed in its most abundant P4-ATPases. In particular, ATP11C was the most abundant P4-ATPase in liver, ATP11A in kidney, ATP8A1 in brain, ATP8A2 and ATP8A1 in retina, and several P4-ATPases including ATP8B3, ATP11C, ATP11B, and ATP11A in testes. Western blots labeled with several well-characterized P4-ATPase antibodies confirmed the results obtained by mass spectrometry. Our proteomic studies serves as a basis for future studies designed to determine the cellular and subcellular distribution of individual P4-ATPases in specific tissues to further define their role in cell physiology and disease.

Previous heterologous cell expression studies have shown that Class 1a (ATP8A1, ATP8A2), Class 5 (ATP10A, ATP10B, ATP10D), and Class 6 (ATP11A, ATP11B, ATP11C) P4-ATPases specifically associate with the CDC50A variant, whereas Class 1b P4-ATPases including ATP8B1, ATP8B2, and ATP8B4 can associate with either the CDC50A or CDC50B β-subunit^12,24–26,29^. Our proteomic studies confirm the association of members of Class 1a, Class 5 and Class 6 P4-ATPases with CDC50A in native tissues. We further show that ATP8B1, ATP8B2, and ATP8B3 also associate with CDC50A in their respective tissues. It is unclear if another fraction of these Class 1b P4-ATPases associate with the CDC50B subunit *in situ*. Proteomic techniques described in this study together with an antibody to CDC50B should be useful in identifying the members of Class 1b P4-ATPases that interact with CDC50B in specific tissues. Likewise, immunoprecipitation studies using antibodies to CDC50C should be instrumental in identifying the P4-ATPase that specifically associates with this CDC50 variant. Identification of CDC50 variants that interact with specific P4-ATPases is an important step in further clarifying their effect on the molecular and cellular properties of these flippases.

The principal function of P4-ATPases is to generate and maintain transbilayer lipid asymmetry by actively transporting specific phospholipids across cell membranes. ATP8A2-CDC50A and ATP8A1-CDC50A complexes were the only mammalian P4-ATPases that had been expressed, purified, and reconstituted in proteoliposomes for direct analysis of their phospholipid flippase activity^25,33,34^. In the present study, we have extended this analysis to ATP11A, ATP11B, and ATP11C, three P4-ATPases widely expressed in tissues. In each case, the ATPase activity of these flippases was stimulated by PS and PE. Importantly, like ATP8A2 and ATP8A1, purified and reconstituted ATP11A, ATP11B, and ATP11C actively transported PS and to a lesser extent PE across membranes. Our studies are in general agreement with earlier cell-based assays which showed that ATP11A and ATP11C import fluorescently-labeled PS and PE into cells^35,42^. We further show here for the first time that ATP11B possesses similar substrate specificities as ATP11A and ATP11C, but exhibit different kinetic properties.

Substrate specificity studies to date indicate that there are two major categories of P4-ATPases, one with a preference for PS > PE (ATP8A1, ATP8A2, ATP11A, ATP11B, ATP11C) and another selective for PC (ATP8B1, ATP8B2, ATP10A). The substrate specificities of ATP8B3, ATP8B4, ATP10B, ATP10D, ATP9A, and ATP9B have yet to be rigorously determined. The reasons why there are so many different P4-ATPases with similar substrate specificities remain to be determined. However, the various P4-ATPases appear to differ in tissue expression and cellular and subcellular localization. They also most likely differ in their regulation, and protein-protein associations, areas that require further studies.

A number of P4-ATPases in our data set have been implicated in important cellular processes and severe diseases. ATP11C, the major P4-ATPase in our dataset in liver, has been previously localized to intracellular vesicles and basolateral membranes of hepatocytes where it plays a critical role in the uptake of unconjugated bile salts by targeting basolateral bile salt transporters to the basolateral membranes^43^. Mice deficient in ATP11C experience multiple abnormalities including hyperbilirubinemia, hepatocellular carcinoma, anemia, X-linked cholestasis and loss in B cell development supporting the importance of this P4-ATPase in liver and blood physiology^7–9^. Our studies show that the ATP8B1-CDC50A complex is also present in liver, but in lower amounts. ATP8B1 has been reported to localize to the canalicular membranes of hepatocytes where its phospholipid flippase activity has been implicated in stabilizing membranes against the effect of bile salts^44–46^. ATP8B1 may also play a role in the expression and localization of the canalicular efflux ABC transporters, ABCB11 (BSEP) and ABCC2 (MRP2). It is possible that an additional fraction of ATP8B1 associates with the CDC50B in hepatocytes, but this remains to be determined *in vivo*. The recent study reporting that mice deficient in liver CDC50A (TMEM30A) exhibit severe intrahepatic cholestasis supports the importance of ATP8B1-CDC50A and ATP11C-CDC50A complexes in bile salt homeostasis^47^.

Testes is a rich source of P4-ATPases. Nine P4-ATPases were identified in our proteomic analysis of mouse testes including ATP8B3 not detected in the other tissues. Although previous heterologous expression studies have failed to identify the CDC50 subunit variant that associates with ATP8B3^24^, our studies clearly show that CDC50A serves as a subunit for ATP8B3. In earlier studies, ATP8B3 was shown to localize to the acrosomal region of spermatozoa^36,48^. Mice deficient in ATP8B3 were fertile, but the average litter size was low and *in vitro* fertilization was suppressed. Furthermore, PS was detected on the outer leaflet before capacitation indicating that ATP8B3 was important in maintaining PS asymmetry. The localization of other P4-ATPases in testes has not been studied in detail.

P4-ATPases are also abundant in neural tissue. In our proteomic screen, we detected seven P4-ATPases in brain and five P4-ATPases in retina. Of the P4-ATPase-CDC50A complexes detected, ATP8A2-CDC50A, in particular, has been shown to play a crucial role in brain and retina. Mice deficient in ATP8A2 are smaller than their wild-type littermates, live only 1–2 months, exhibit severe neurological abnormalities including ataxia, body tremors, distal axonal degeneration of spinal neurons, and display sensory defects associated with reduced function and degeneration of photoreceptor cells and ganglion cells of the retina and spiral ganglion cells of the auditory system^5,6^. In humans, a loss-in-function mutation in *ATP8A2* is responsible for CAMRQ syndrome, a severe disease characterized by cerebellar ataxia, mental retardation, and dysarthric speech with or without quadrupedal gait^14,19^. Mutations in *ATP8A2* have also been linked to encephalopathy, intellectual disability, severe hypotonia, chorea and optic nerve atrophy in patient populations^15^. In contrast, mice deficient in ATP8A1, a related P4-ATPase present in relatively high levels in the brain as shown here, displayed a milder phenotype^49^. ATP8A1 knockout mice had a normal appearance, but were hyperactive and exhibited a deficiency in hippocampus-dependent learning. ATP8A1 and ATP8A2, which share a 67% identity in amino acid sequence, exhibit some redundancy in function, since mice deficient in both flippases die just after birth^5^. In the present study we compared the localization of ATP8A1 with ATP8A2 in the retina. Unlike ATP8A2, ATP8A1 was undetectable in photoreceptor outer segments, but was present with ATP8A2 in photoreceptor inner segments suggesting these flippases may play an overlapping role in generating PS and/or PE asymmetry important in vesicle trafficking within photoreceptors.

In conclusion our proteomic analysis has identified ten P4-ATPases in various tissues that utilize CDC50A as their β-subunit. This information provides a foundation for further defining the cellular and subcellular localization of these phospholipid flippases for elucidating their role in cell physiology and disease. Biochemical analyses of purified ATP11-CDC50A complexes identified PS and PE as substrates for ATP11B and confirmed previous cell based assays showing that ATP11A and ATP11C transport these same substrates.

## Methods

### Materials

Unlabeled 1,2 dioleoyl-sn-glycero-3-phosphocholine (DOPC), 1,2 dioleoyl-sn-glycero-3-phosphoserine (DOPS), 1,2 dioleoyl-sn-glycero-3-phosphoethanolamine (DOPE), L-α-phosphatidylcholine (PC) (egg, chicken), porcine brain polar lipids (BPL), and NBD-label phospholipids: 1-oleoyl-2-[7-nitro-2-1,3-benzoxadiazol-4-yl] (NBD) hexanoyl phospholipids: NBD-PC; NBD-PE; NBD-PS were obtained from Avanti Polar Lipids (Alabaster, AL). Synthetic 1D4 (TETSQVAPA) and 7F4 (Ac-AKDEVDGGP) peptides were from Biomatik (Cambridge, ON), restriction enzymes from New England Biolabs (Ipswich, MA), porcine-modified trypsin from Promega (Nepean, ON), and endopeptidase Lys-C from Wako Chemicals (Osaka, Japan). Antibodies were obtained from the following sources: rabbit ATP8A1 antibody from Proteintech, monoclonal mouse Rho1D4 antibody through Flintbox (http://www.rho1d4.com/), anti-ATP11A from Santa Cruz, and secondary anti-mouse and anti-rabbit Ig antibodies conjugated to IRDye 800 dye from Li-Cor Biosciences. Polyclonal antibody to mouse ATP8A2 and monoclonal antibody to CDC50A (Cdc50-7F4) have been previously described^6,25^. Monoclonal antibody ATP11C-11E3 was generated from mice immunized with a GST-fusion protein containing mouse ATP11C residues 429–500 using established monoclonal antibody generating techniques. Purified Rho 1D4 and CDC50A monoclonal antibodies were directly coupled to CNBr-activated Sepharose 2B at 1.5 mg antibody per ml of beads as previously described^25,50^.

### Identification of P4-ATPase-CDC50A complexes in mouse tissues by mass spectrometry

Live animals were not used in these studies. Euthanasia of mice was approved by the UBC Institutional Animal Care Committee. All methods were carried out in accordance with relevant institutional guidelines and regulations of UBC. Slices of adult mouse tissues (retina, brain, liver, testes and kidney) were incubated in 10 mM Tris, pH7.4, 0.075 mM NaCl, and Protease Arrest for 1 h on ice. The extracts were added to 1 volume of buffer consisting of 40 mM Tris, pH7.4, 0.15 M NaCl, and 17% w/v sucrose and homogenized in a Dounce homogenizer. The homogenate was layered on a 52% w/v sucrose cushion and centrifuged for 1 h at 28,000 rpm in a SW 28 swing bucket rotor. The membranes were collected on top of the 52% (w/v) sucrose, diluted 3 fold in 40 mM Tris buffer, pH 7.4, and centrifuged at 40,000 rpm for 20 min. The pellet was solubilized in 18 mM CHAPS, 40 mM Tris, pH 7.4, 150 mM NaCl, 5 mM MgCl_2_, and 1 mM DTT for 1 h at 4 °C with stirring to obtain a protein concentration of 2 mg/ml. The solution was centrifuged at 40,000 rpm for 20 min. Ten ml of the solubilized extract (20 mg protein) was incubated at 4 °C for 1 h with 75 µl of Cdc50-7F4-Sepharose pre-equilibrated in solubilization buffer, and subsequently washed 6 times with 500 µl of buffer consisting of 10 mM CHAPS, 40 mM Tris, pH 7.4, 150 mM NaCl, 5 mM MgCl_2_ and 1 mM DTT. The bound protein was eluted in the same buffer containing 0.2 mg/ml of 7F4 peptide.

For in gel digestion, the eluted samples were subjected to SDS gel electrophoresis allowing the proteins to just enter the separating gel. In-gel trypsin digestion of the gel-slice was performed as previously described^51^. Alternatively, the eluted samples were digested with endopeptidase Lys-C followed by trypsin using the filter-aided sample preparation (FASP) of Wisniewski *et al*.^39^.

In either case the trypsin digested samples were submitted to the Proteomics Core Facility of Michael Smith Laboratories (MSL) at the University of British Columbia for the mass spectrometric analysis. Briefly, the samples were acidified with 1% TFA and desalted using a C18 STAGE-tip. Digested peptides were analyzed by a quadrupole–time of flight mass spectrometer (Impact II; Bruker Daltonics) coupled to an Easy nano LC 1000 HPLC (ThermoFisher Scientific) using an analytical column that was 40–50 cm long, with a 75-μm inner diameter fused silica with an integrated spray tip pulled with P-2000 laser puller (Sutter Instruments), packed with 1.9 μm diameter Reprosil-Pur C-18-AQ beads (Maisch, www.Dr-Maisch.com), and operated at 50 °C with in-house built column heater. Buffer A consisted of 0.1% aqueous formic acid, and buffer B consisted of 0.1% formic acid and 80% (vol/vol) acetonitrile in water. A standard 60-min peptide separation was done, and the column was washed with 100% buffer B before re-equilibration with buffer A. The Impact II was set to acquire in a data-dependent auto-MS/MS mode with inactive focus fragmenting the 20 most abundant ions (one at the time at 18-Hz rate) after each full-range scan from m/z 200 to m/z 2,000 at 5 Hz rate. The isolation window for MS/MS was 2–3 depending on the parent ion mass to charge ratio, and the collision energy ranged from 23 to 65 eV depending on ion mass and charge. Parent ions were then excluded from MS/MS for the next 0.4 min and reconsidered if their intensity increased more than five times. Singly charged ions were excluded from fragmentation.

Data were searched using MaxQuant (v1.5.3.30)^52^. The search was performed against a database comprised of the protein sequences from Uniprot’s mouse entries plus common contaminants with variable modifications of methionine oxidation, and N-acetylation of the proteins. Only those peptides exceeding the individually calculated 99% confidence limit (as opposed to the average limit for the whole experiment) were considered as accurately identified. Three independent samples of retina tissue were analyzed and yielded peptides that identified the same five P4-ATPases and CDC50A.

The mass spectrometry proteomics data have been deposited to the ProteomeXchange Consortium via the PRIDE partner repository with the dataset identifier PXD009143. Summary of the most abundant proteins is given in the Supplemental Materials (Tables S1–S5).

### Generation of ATP11A, ATP11B, ATP11C constructs

The cDNA encoding human ATP11B (NM 014616) in pCAG-HAC plasmid was kindly provided by Dr. Hye-Won Shin (Kyoto University). The cDNA encoding human ATP11A was purchased from DNASU (plasmids ID: HsCD00295091; https://dnasu.org/DNASU/SearchTemplate.do) and the mouse cDNA for ATP11C was from Open Biosystems (IMAGE:30843359). The three ATP11 constructs containing the 1D4 epitope tag were cloned into pcDNA3 by polymerase chain reaction (PCR) using primers with the appropriate restriction sites (BamHI/XbaI for *ATP11A*; XhoI/XbaI for *ATP11B*; BamH1/XbaI for *ATP11C*). Constructs containing point mutations were subsequently generated using QuikChange site-directed mutagenesis according to the manufacturer’s instructions (Agilent Technologies). The identity of all the constructs and mutations were confirmed by Sanger DNA sequencing.

### Cell culture and transfection

HEK293T cells (American Type Tissue Collection, Manassas, VA) grown in Dulbecco’s Modified Eagle Media (DMEM) containing 10% fetal bovine serum (FBS), 100 μg/ml streptomycin, 100 units/ml penicillin, and 2 mM L-glutamine were co-transfected in 10 cm dishes with 10 µg of pcDNA3-*ATP11* and 10 µg of pcDNA3-*CDC50A* per dish by the calcium phosphate method as previously described^25^ and harvested 24–48 h after transfection. For some studies, 20 µg of the mouse *ATP11C* cDNA was co-transfected with 20 µg of plasmid DNA containing *CDC50A* to increase the expression of the ATP11C-CDC50A complex.

### Purification of ATP11A, ATP11B and ATP11C from HEK293T cells

ATP11 proteins harboring a 9 amino acid C-terminal 1D4 tag (TETSQVAPA) were co-expressed with CDC50A in HEK293T cells and purified on a Rho1D4 immunoaffinity matrix as previously described^25^. Briefly, 1 ml of Buffer A (50 mM HEPES, pH 7.5, 150 mM NaCl, 5 mM MgCl_2_, 1 mM DTT, 20 mM CHAPS, 0.5 mg/ml egg phosphatidylcholine (PC), and cOmplete inhibitor) was added to HEK293T cells in each of 10 culture dishes (10 cm dia.). After 30 min, the insoluble material was removed by centrifugation at 100,000 × g for 10 min. The detergent-solubilized fraction was incubated at 4 °C with approximately 30 µl of packed immunoaffinity matrix (Rho 1D4 antibody coupled to CNBr-activated Sepharose) pre-equilibrated in Buffer A. After 2 h, the matrix was washed 6 times with 500 μl Buffer B (50 mM HEPES, pH 7.5, 150 mM NaCl, 5 mM MgCl_2_, 1 mM DTT, 10 mM CHAPS, 0.75% (w/v) octyl glucoside, 0.5 mg/ml DOPC) and the ATP11-CDC50A complex was subsequently eluted twice for 30 min each at 23 °C with 50 µl of Buffer B containing 0.2 mg/ml of 1D4 peptide. Approximately, 5 µg of the purified complex in 100 µl of Buffer B was typically obtained.

### Reconstitution of ATP11-CDC50A complexes for functional characterization

Five µg of immunoaffinity purified complex in 100 µl of Buffer B was mixed with an equal volume of reconstitution buffer (50 mM HEPES, pH 7.5, 150 mM NaCl, 5 mM MgCl_2_, 1 mM DTT, 1% octyl glucoside, 10% sucrose and 2.5 mg/ml of the designated phospholipid). The designated phospholipid was brain polar lipid (BPL), 100% DOPC, or 90%DOPC and 10% lipid specified in the experiment and typically DOPS or DOPE. The mixture with a lipid to protein ratio of 50:1 was stirred for 30 min at room temperature and subsequently dialyzed at 4 °C against 1 l of dialysis buffer (10 mM HEPES-NaOH pH 7.5, 150 mM NaCl, 5 mM MgCl_2_, 1 mM DTT, 10% sucrose) for 16–20 h with 2–3 changes of buffer to remove the detergent and generate the proteoliposomes for ATPase and phospholipid flippase assays. In some flippase experiments, egg phosphatidylcholine PC was substituted for synthetic DOPC although this difference in phospholipids did not affect the flippase activity.

### ATPase activity assays

For ATPase assays, approximately 25 ng of reconstituted protein was added to 25 µl of buffer containing 50 mM HEPES-NaOH, pH 7.5, 150 mM NaCl, 12.5 mM MgCl_2_, 1 mM DTT, 10% sucrose, 10 mM CHAPS and the designated concentration of ATP. Samples were incubated at 37 °C for 60 min and stopped by the addition of an equal volume of 12% SDS. The concentration of phosphate product was determined by a colorimetric microtiter plate assay as previously described^33^. The amount of phosphate released was determined from a standard curve generated from known phosphate concentrations. For inhibitor studies, the proteoliposomes (90% DOPC/10%DOPS) were treated at a final concentration with 100 μM NaF, 100 μM vanadate, 5 mM N-ethylmaleimide (NEM), or 1 mM ouabain prior to the addition of ATP for ATPase measurements. Samples containing all the components except the proteoliposomes served as background measurements. Similar measurements were obtained when liposomes without the P4-ATPase complex were used as background controls. All data points were performed in triplicate and each experiment repeated at least 3 times with similar results. Kinetic data and statistics were analyzed using Graph-Pad Prism 7.

### Phospholipid flippase assays

The flippase assay was carried out as previously described^25,33^, with several modifications. P4-ATPases (~ 0.2 µg) were reconstituted into liposomes consisting of 97.5% egg PC or DOPC and 2.5% (w/w) NBD-phospholipid as described above at a phospholipid to protein ratio of 50:1. The proteoliposomes were mixed with either 1 mM ATP or AMP-PNP (control) in 10 mM HEPES, pH 7, 150 mM NaCl, 5 mM MgCl_2_, 1 mM DTT, and 10% sucrose in 20 µl. After 2.5 min, 180 µl of 10 mM HEPES pH 7, 150 mM NaCl, 10 mM EDTA, 1 mM DTT, 10% sucrose was added to stop the transport reaction. The reaction was linear at this time point (Supplemental Fig. S-2). NBD-fluorescence was typically measured in a microtiter plate reader (SpectraMax M3, Molecular Devices) using a 96-well black wall, clear bottom microtiter plate (Corning). Dithionite was added at a final concentration of 2 mM to bleach the NBD-phospholipids on the outer leaflet. After the fluorescence stabilized (~7.5 min), 1% Triton X-100 was added to bleach the remaining NBD-phospholipids on the inner leaflet of the proteoliposomes. A typical fluorescence profile is shown in supplemental material (Fig. S-1). Similar bleaching profiles have been previously reported for fluorescent phospholipid assays^18,25,33,53^. The percentage of NBD-PL present on the outside of the liposomes was calculated using the following formula %NBDout = (NBDi - NBDd)/NBDi x 100% where NBDi is the initial fluorescence and NBDd is the fluorescence following dithionite addition. The %NBD-PL flipped was determined from the difference in the %NBDout for the sample containing ATP and the sample containing AMP-PNP and normalized to the protein amount of the P4-ATPase assayed. Data was an average of three independent experiments.

### Data availability statement

All source data for figures and tables in the present article are available from the corresponding author on reasonable request.

### SDS gels and Western blotting

Protein samples were resolved by SDS-gel electrophoresis on 8% acrylamide gels and either stained with Coomassie blue or transferred to Immobilon FL membranes (Millipore, Bedford, MA). The concentration of purified P4-ATPases (ATP11A, ATP11B, and ATP1C) was determined on Coomassie blue stained SDS gels using bovine serum albumin as a standard. For western blotting, membranes were blocked with 1% milk in PBS for 30 min and subsequently incubated with the primary antibody in blocking buffer for 1 h. After three washes in phosphate buffered saline (PBS) containing 0.05% Tween 20 (PBST), the membranes were incubated with a secondary goat anti-rabbit or goat anti-mouse Ig antibody conjugated with IRDye 800 dye (Li-Cor Biosciences) diluted 1:20,000 in blocking buffer for 30 min and subsequently washed with PBST three times. The primary antibodies used for western blots were diluted as follows: ATP11C-11E3 and Cdc50-7F4 hybridoma cell culture fluid diluted 1:10 in PBS; Rho1D4 hybridoma cell culture fluid diluted 1:100; ATP11A and ATP8A1 were diluted as recommended by the manufacturers. Labeled membranes were visualized on an Odyssey Infrared Imaging System (LI-COR Biosciences).

### Immunofluorescence microscopy

Cryosections of adult mouse retinal tissues were fixed with 4% paraformaldehyde in 100 mM phosphate buffer (PB, pH 7.4) for 60 min as previously described^54^. The sections were subsequently blocked, and permeabilized with PB containing 0.2% Triton X-100 and 10% normal goat serum for 30 min and labeled overnight at room temperature with antibodies to ATP8A2 and ATP8A1 diluted 1:200 in PB containing 2.5% normal goat serum and 0.1% Triton X-100. Sections were washed 3 times with PB and labeled for 3 h with goat anti-rabbit Alexa-594 secondary antibody (diluted 1:1000) and counterstained with DAPI nuclear stain. The sections were examined on a Zeiss LSM 700 confocal microscope with a Zen imaging system.

## Electronic supplementary material

Supplemental Material
