# Supplementary material for: Proteomic Analysis and Functional Characterization of P4-ATPase Phospholipid Flippases from Murine Tissues
Source: Sci Rep. 2018 Jul 17;8:10795. doi: 10.1038/s41598-018-29108-z (PMC6050252; doi:10.1038/s41598-018-29108-z)
Supplement: Supplementary file 1 — Supplemental Material [file 41598_2018_29108_MOESM1_ESM.pdf]

Supplemental Material

Proteomic Analysis and Functional Characterization of P4-ATPase Phospholipid Flippases from Murine Tissues

Jiao Wang[1,2], Laurie L. Molday[1], Theresa Hii[1], Jonathan. A. Coleman[1], Tieqiao Wen[2], Jens P. Andersen[3], Robert S. Molday[1,*]

Supplemental Figures S1 and S2

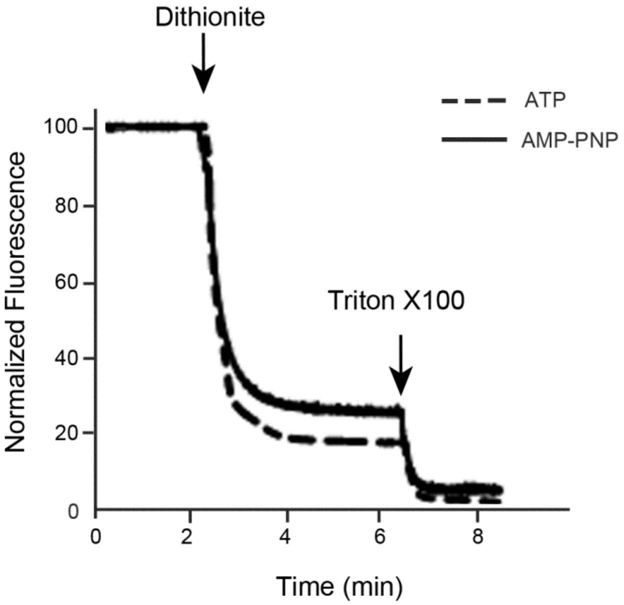

Figure S1: Typical fluorescence bleaching profile for phospholipid flippase measurements. ATP11A-CDC50A complex reconstituted into liposomes consisting of 97.5% DOPC and 2.5% NBD-PS was incubated at 37 ºC for 2.5 min with either 1 mM ATP or 1 mM AMP-PNP as a control. The fluorescence was monitored at room temperature. As indicated, dithionite (2 mM) was added to bleach fluorescent lipids on the outer leaflet of the liposomes. After the fluorescence stabilized, 1% Triton X-100 was added to bleach the remaining fluorescent lipids on the inner leaflet.

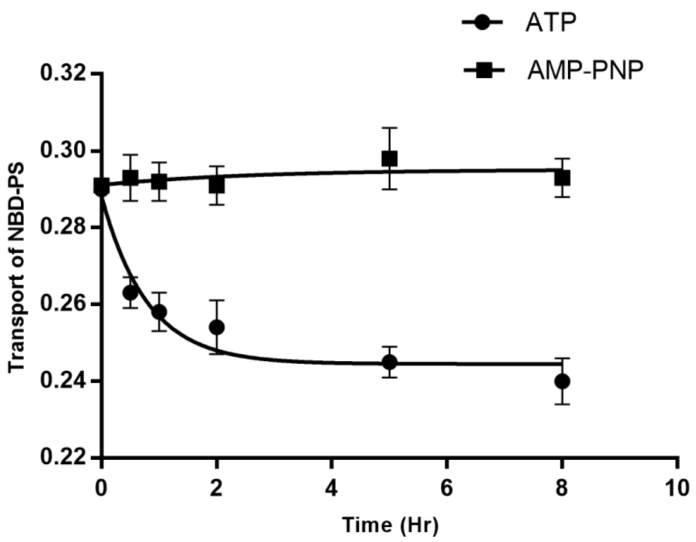

Figure S-2: Time course for transport of NBD-PS from the inner to the outer leaflet of proteoliposomes containing ATP11A-CDC50A complex. Proteoliposomes were incubated with 1 mM ATP or AMP-PNP (control) at 37°C. At various times indicated, the reaction was stopped by the addition of buffer containing EDTA. The amount of NBD-PS was determined after fluorescence bleaching by dithionite as indicated in the Methods section. No transport was observed when ATP was substituted with AMP-PNP.

Supplemental Tables

Supplemental Tables S1 – S6;

Table S1 Summary of Abundant Proteins from Retina that Eluted from a Cdc7F4 immunoaffinity matrix.

Table S2 Summary of Abundant Proteins from Brain that Eluted from a Cdc7F4 immunoaffinity matrix.

Table S3 Summary of Abundant Proteins from Liver that Eluted from a Cdc7F4 immunoaffinity matrix.

Table S4 Summary of Abundant Proteins from Testes that Eluted from a Cdc7F4 immunoaffinity matrix.

Table S5 Summary of Abundant Proteins from Kidney that Eluted from a Cdc7F4 immunoaffinity matrix.

Table S6 Control example: Summary of Abundant Proteins from Retina that Eluted from a Myc immunoaffinity matrix.

Table S-1 Abundant Proteins From Mouse Retina

Protein IDs	Protein names	Gene name	Peptides SF	Mol. weigh	Sequence	Sequence coverage SP	Intensity
P98200;D6	Probable phospholipid-tr	Atp8a2	33	129.42	1148	32.1	1480400000
P70704;F6	Probable phospholipid-tr	Atp8a1	31	129.77	1149	32.3	1192300000
Q68FD5;Q	Clathrin heavy chain 1	Cltc	35	191.55	1675	26	632080000
P68372	Tubulin beta-4B chain	Tubb4b	15	49.83	445	47.6	433540000
Q6DFW5;Q6ZQ17;E9Q0M7;F6TYE6		Atp11b	22	133.53	1175	23.7	406570000
Q792Z1		Try10	1	26.221	246	4.1	390600000
Q3UV17	Keratin, type II cytoskele	Krt76	4	62.844	594	4.7	354840000
P21981;G3	Protein-glutamine gamm	Tgm2	20	77.06	686	37.9	322610000
P15105;D3	Glutamine synthetase	Glul	12	42.119	373	39.1	304510000
P01630	Ig kappa chain V-II region	7S34.1	2	12.496	113	21.2	292930000
Q5FW97;P	Alpha-enolase;Enolase	Eno1	11	47.14	434	38	263020000
Q545A2;P5	ADP/ATP translocase 2	Slc25a5	8	32.931	298	28.5	248960000
P52480;P5	Pyruvate kinase isozyme	Pkm	18	57.844	531	39.9	197130000
F7CES2;F7C583;Q3UX37;F6S200		Plekhg1	1	16.665	150	10	143930000
P16858;E9	Glyceraldehyde-3-phospl	Gapdh;Gm	6	35.81	333	24.3	140440000
P99024	Tubulin beta-5 chain	Tubb5	14	49.67	444	44.6	140440000
P15409	Rhodopsin	Rho	3	39.069	348	11.5	129590000
Q58E64;P1	Elongation factor 1-alpha	Eef1a1;Eef	7	50.113	462	15.6	124640000
P63017;Q5	Heat shock cognate 71 kl	Hspa8	12	70.87	646	23.5	121710000
Q6PIC6;Q8	Sodium/potassium-trans	Atp1a3	21	111.69	1013	24.7	118120000
P01631;F6	Ig kappa chain V-II regior	Igkv1-115	1	12.273	113	11.5	107250000
P48962;Q3	ADP/ATP translocase 1	Slc25a4	7	32.904	298	24.5	99107000
F6U8D7;Q	Probable phospholipid-tr	Atp11c	11	127.15	1111	12.2	95995000
P98197;E9	Probable phospholipid-tr	Atp11a	16	135.5	1187	15.1	94860000
Q71LX8;P1	Heat shock protein HSP	Hsp90ab1	11	83.28	724	18.4	82287000
P56480	ATP synthase subunit be	Atp5b	9	56.3	529	23.6	82051000
Q8VEK0;D	Cell cycle control protein	Tmem30a	7	41.06	364	22.5	77911000
P20612;Q3	Guanine nucleotide-bind	Gnat1	9	39.966	350	32.3	75332000
P01801;P0	Ig heavy chain V-III region	J606;Ig he	2	12.81	115	20.9	72221000
Q564E2;P0	L-lactate dehydrogenase	Ldha	8	36.498	332	26.5	67336000
Q61301-2;	Catenin alpha-2	Ctnna2	13	100.42	905	17.9	65545000
P01868;P0	Ig gamma-1 chain C regic	Ighg1	3	35.704	324	14.8	58649000

P68369;Q5	Tubulin alpha-1A chain;T	Tuba1a;Tul	14	50.135	451	42.1	56894000
Q3UPX6;P2	S-arrestin	Sag	6	44.93	403	20.6	56015000
Q03265;D3	ATP synthase subunit alp	Atp5a1	5	59.752	553	12.7	51934000
Q8BHD8;B(	Protein-L-isoaspartate O-	Pcmtd2	6	40.755	359	18.7	51767000
Q9Z1R9		Prss1	1	26.134	246	8.1	50976000
Q02248;E9	Catenin beta-1	Ctnnb1	8	85.47	781	10.9	36931000
F8WIX8;Q8	Histone H2A;Histone H2/	Hist1h2al;H	2	13.607	125	20.8	36482000
P01837	Ig kappa chain C region		2	11.778	106	26.4	35500000
G3X9J7;G5	Fibronectin;Anastellin	Fn1	7	253.01	2296	3.8	33755000
F7CAE1;Q9	Hemoglobin subunit alph	Hba-a1;Hba	2	14.932	140	17.9	32287000
A8DUK4;E9	Hemoglobin subunit beta	Hbb-b1;Hb	4	15.748	147	34	31406000
Q561N5;P6	40S ribosomal protein S1	Rps18;Rps1	5	17.718	152	31.6	30176000
G3XA10;Q8	Heterogeneous nuclear r	Hnrnpu	5	86.805	793	10	29421000
P62806;B2	Histone H4	Hist1h4a;G	2	11.367	103	19.4	28747000
Q04447	Creatine kinase B-type	Ckb	5	42.713	381	15.7	27706000
Q3UWK3;P	Peripherin-2	Prph2	4	39.259	346	20.5	26610000
Q8VEM8;G	Phosphate carrier protei	Slc25a3	4	39.632	357	11.5	24613000
Q5FWB7;P(	Fructose-bisphosphate a	Aldoa;Aldo	4	39.355	364	13.5	24269000

Table S2 Abundant Proteins from Mouse Brain

Protein IDs	Peptide cou	Protein names	Gene name	Number of	Peptides	Sequence coverag	Mol. weigh	Sequence l	Intensity
A3KGU7;P1	83;82;81;8:	Spectrin alpha chain, non-er	Sptan1	7	83	41.6	285.15	2477	25098000
F6WYQ5;P	78;78;77;1!	Phospholipid-transporting A	Atp8a1	7	78	51.2	131.28	1163	616030000
Q62261;Q(	64;60	Spectrin beta chain, non-ery	Sptbn1	2	64	34.7	274.22	2363	18963000
E0CXB9;Q6	58;58;57;5	Catenin alpha-2	Ctnna2	5	58	54.8	106.73	966	157690000
Q5SXR6;Q(	56;56;7	Clathrin heavy chain;Clathrir	Cltc	3	56	40.3	191.98	1679	34840000
F6Q8D3;Q!	46;46	Phospholipid-transporting A	Atp11c	2	46	38.2	129.11	1129	140080000
E9Q3L2	45;8;4;3;3		Pi4ka	5	45	28.6	231.35	2044	11399000
P98200	42;6;6;1	Phospholipid-transporting A	Atp8a2	4	43	34.7	129.42	1148	143880000
Q6DFW5;E	39;36	Phospholipid-transporting A	Atp11b	2	42	34.9	133.53	1175	93389000
E9Q3G7;P!	36;35;16;6;	Phospholipid-transporting A	Atp11a	7	40	33.5	130.68	1142	90790000
F6TYE6	2			1	38	38.6	110.11	967	665050
P20029	31	78 kDa glucose-regulated pr	Hspa5	1	31	47	72.421	655	28368000
Q9JHU4	31	Cytoplasmic dynein 1 heavy	Dync1h1	1	31	10.1	532.04	4644	2981200
Q02248;E9	28;20;7;7;4	Catenin beta-1	Ctnnb1	9	28	45.6	85.47	781	101600000
Q8VEK0;D:	27;24;2	Cell cycle control protein 50.	Tmem30a	3	27	49.7	41.06	364	212960000
P55288	26;7;6;5;2;:	Cadherin-11	Cdh11	6	26	40.1	88.111	796	18314000
D3YYN7;Q(	24;24;14;4;	Sodium/potassium-transpor	Atp1a2	5	24	31.9	103.58	947	22891000
Q6PIC6;Q8	11;10	Sodium/potassium-transpor	Atp1a3	2	23	28.5	111.69	1013	10121000
Q8VDN2	15;0;0;0	Sodium/potassium-transpor	Atp1a1	4	23	27.1	112.98	1023	4127500
P46460	22;3;1	Vesicle-fusing ATPase	Nsf	3	22	30.5	82.613	744	5430600
E9Q6P5	21		Ttc7b	1	21	33.7	94.202	843	16840000
Q504P4;P6	20;20;5;4;3	Heat shock cognate 71 kDa		Hspa8	8	20	45.3	68.778	627	17160000
D3YYT0;P1	19;19	Cadherin-2	Cdh2	2	19	29	93.856	849	41438000
P70408;H3	19;16	Cadherin-10	Cdh10	2	19	26.9	88.311	788	14953000
P63260;P6	18;18;13;1:	Actin, cytoplasmic 2;Actin, c	Actg1;Actb	12	18	49.6	41.792	375	75045000
P99024	18	Tubulin beta-5 chain	Tubb5	1	18	48	49.67	444	55425000
Q03265;D3	18;17;2	ATP synthase subunit alpha,	Atp5a1	3	18	43.2	59.752	553	13922000
Q9D6F9	10	Tubulin beta-4A chain	Tubb4a	1	18	48	49.585	444	11366000
Q9ERD7	9	Tubulin beta-3 chain	Tubb3	1	18	43.3	50.418	450	5622800
O08599-2;(	18;17	Syntaxin-binding protein 1	Stxbp1	2	18	43.3	68.735	603	5072400
Q8C8R3-2;	18;16;16;1	Ankyrin-2	Ank2	33	18	6.3	428.76	3926	3099700
F8WHB1;Q	18;18	Calcium-transporting ATPase	Atp2b2	2	18	19.1	136.78	1243	3085600

P04370-4;F	17;16;16;1(	Myelin basic protein	Mbp	10	17	67.2	21.502	195	41686000
P68369;P0.	17;14		Tubulin alpha-1A chain;Tubu	Tuba1a;Tul	2	17	38.8	50.135	451	30662000
P16330-2;F	17;17		2,3-cyclic-nucleotide 3-phos	Cnp	2	17	49.2	44.654	400	16515000
P46096	17;6		Synaptotagmin-1	Syt1	2	17	39	47.417	421	7769300
P68373;P0.	1;1;1;0		Tubulin alpha-1C chain;Tubu	Tuba1c;Tul	4	17	39	49.909	449	1932300
Q7TMM9	6;0		Tubulin beta-2A chain	Tubb2a	2	16	47.9	49.906	445	15066000
P56480		16	ATP synthase subunit beta, r	Atp5b	1	16	50.5	56.3	529	13755000
Q02257		15	Junction plakoglobin	Jup	1	16	26	81.8	745	7160700
G3UVV4;P1	16;16;16;1(		Hexokinase;Hexokinase-1	Hk1	11	16	20.3	101.87	917	3562100
P50516;P5(	16;11;4;3		V-type proton ATPase cataly	Atp6v1a	4	16	34.8	68.325	617	3271000
P12960		16	Contactin-1	Cntn1	1	16	19	113.39	1020	2583400
P68368		4	Tubulin alpha-4A chain	Tuba4a	1	16	35.3	49.924	448	2359600
P68372;J3(	1;1		Tubulin beta-4B chain	Tubb4b	2	16	47	49.83	445	396380
G5E829		6	Plasma membrane calcium-t	Atp2b1	1	15	15	134.75	1220	1412300
Q9CWF2		1	Tubulin beta-2B chain	Tubb2b	1	15	47.9	49.953	445	531860
E9Q800;Q8	14;14;14;1		MICOS complex subunit Mic	Immt	9	14	27	75.6	679	3745700
E9QAL4;D3	14;14;14;3;		Phospholipid-transporting A	Atp8b2	6	14	18.2	134.83	1190	3026600
E9QKH8;O3	14;14;14;1		Catenin delta-2	Ctnnd2	4	14	14.4	134.94	1246	2600900
Q8K2X1-2;(	14;14		Probable phospholipid-trans	Atp10d	2	14	13.9	156.34	1400	2526000
P04370-6;F	1;1;1;1;0;1;		Myelin basic protein	Mbp	8	14	68.2	17.225	154	2381100
O08553	14;2;2		Dihydropyrimidinase-relatec	Dpysl2	3	14	35.7	62.277	572	1746000

Table S3 Proteins from Mouse Liver A - Peptides from FASP; B - Peptides from Gel Extraction

Protein IDs	Gene names	Peptides A (FASP)	Peptides B (Gel-Ex)	Mol. weight [kDa]	Seq. length	Seq. coverage A [%]	Seq. coverage B [%]	Intensity A	Intensity B
E9QKK8;Q9QZW0-2	Atp11c	114	30	127.81	1116	65	24.8	1.76E+09	3.6E+08
Q8VEK0;D3YVV1;Q8BHG3	Tmem30a	24	12	41.06	364	60.7	25.8	3.3E+08	1.39E+08
Q8C196;E9QAI5;G3UWN2	Cps1	105	16	164.62	1500	74.7	11.7	6.47E+08	18375000
P20029	Hspa5	41	7	72.421	655	61.5	10.5	79366000	7613200
Q8BMS1	Hadha	38	6	82.669	763	54	8.7	58599000	6559200
P63260;P60710;E9Q1F2;E9Q5F4	Actg1;Actb	18	7	41.792	375	72.3	18.1	87785000	6216300
Q8CAQ8-2;Q8CAQ8;Q8CAQ8-5	Immt	35	10	82.928	746	59	15.5	31803000	4302800
P38647	Hspa9	33	8	73.46	679	59.2	12.8	65926000	4198500
G5E8R3;E9QPD7;Q05920	Pcx;Pc	50	8	129.7	1178	53.9	7.7	74582000	4012100
P25688;D3Z4U1	Uox	20	3	35.039	303	65.7	14.5	1.05E+08	3909500
O35488;A2ANX6	Slc27a2	23	4	70.422	620	52.9	6	28046000	3020000
P47962;D3YYV8	Rpl5	13	5	34.4	297	45.1	18.9	31088000	2897200
O35129;F6QPR1;F6Q8V7	Phb2	19	4	33.296	299	70.6	13	56211000	2616800
Q5SXR6;Q68FD5;F6Z1R4	Cltc	48	5	191.98	1679	42	3.6	32328000	2607700
P26043;Q7TSG6;P26040	Rdx	20	5	68.542	583	40	9.4	10970000	2581100
Q148W0;E9QAL4;A2ANX3	Atp8b1	29	5	143.8	1251	31.2	4.2	18832000	2442400
P51660	Hsd17b4	31	10	79.481	735	54.1	13.7	26235000	2168200
P35486;P35487	Pdha1	13	5	43.231	390	29.2	12.6	8335600	2166400
P24270;A2AL20	Cat	36	4	59.795	527	77.6	9.7	60571000	2149600
Q03265;D3Z6F5;D6RJ16	Atp5a1	46	4	59.752	553	71.1	5.8	1.24E+08	2139600
G5E8D1;A9JTY7;O70562	Sprr2k;Sprr2b	0	1	7.559	68	0	13.2	0	2120400
Q63886	Ugt1a1	19	2	60.047	535	42.6	4.1	34972000	2098700
P63038;P63038-2;D3Z2F2;D3Z7J9	Hspd1	34	5	60.955	573	68.1	8	73734000	2092400
P26443;F7CFA5	Glud1	33	2	61.336	558	67.6	3.4	77141000	1967500
P63017;Q504P4;P17156;D3Z5E2;D3YW43	Hspa8	16	5	70.87	646	37.3	8.7	8929500	1957900
Q8BWT1	Acaa2	29	4	41.829	397	81.9	14.6	1.42E+08	1939200
P51881;P48962;Q3V132	Slc25a5	23	2	32.931	298	64.8	6	43029000	1914700
P35564	Canx	9	4	67.277	591	25.7	8.3	5580100	1905400

P53395	Dbt	13	5	53.246	482	41.3	9.5	17619000	1875400
E9Q3G7;P98197	Atp11a	30	7	130.68	1142	33.8	7.5	17997000	1852300
P08101-2;E9Q415	Fcgr2;Fcgr2b	8	4	31.881	283	25.1	17.3	16921000	1844400
P08113;F7C312	Hsp90b1	31	5	92.475	802	43.3	6.5	27223000	1789500
Q8VEM8;G5E902	Slc25a3	17	4	39.632	357	50.4	10.4	21599000	1782700
Q9DBG1	Cyp27a1	21	4	60.719	533	46.5	8.8	17436000	1767500
A3KGU5;A3KGU7	Sptan1	43	10	282.89	2457	24.6	5.5	13384000	1732000
P97872;Q14DT3;Q8C116	Fmo5	23	4	60	533	50.8	8.4	29234000	1719200
P55096	Abcd3	27	5	75.474	659	46.3	7.3	25168000	1707400
Q99MN9;E9Q1J7;D3YZC1	Pccb	11	3	58.408	541	30.3	7.9	10056000	1639900
B7ZCM8;B7ZCN0;B7ZCM9;P0C871	Pla2g4b;Gm280	1	1	89.455	791	1	1	180110	1636200
Q60634;Q60634-3	Flot2	38	6	47.037	428	82	12.9	1.63E+08	1588100
Q8R084	Ugt2b1	16	3	60.157	529	43.3	7.6	30617000	1566600
P97742;Q924X2	Cpt1a	13	4	88.25	773	21.5	5.8	6659800	1557800
Q9CQQ7	Atp5f1	12	4	28.948	256	44.5	16.8	33190000	1530700
B1AQR8;G3X9T7;O08573-2;O08573	Lgals9	12	5	39.907	352	34.4	17.6	22945000	1521300
Q9JJL3;Q9JJL3-2	Slco1b2	5	2	76.728	689	12.2	3.5	4346700	1462900
Q02257	Jup	20	2	81.8	745	37.3	3.6	21143000	1405000
P01942;Q91VB8;A7M7S6;P06467	Hba;haemaglobi	10	2	15.085	142	86.6	15.5	1.2E+08	1383400
E9QAZ2;Q9CZM2;E9Q1X1;B8JKK2	Gm10020;Rpl15	11	3	24.077	204	43.1	17.6	10718000	1372200
O08917;G3UYU4	Flot1	44	6	47.513	428	84.3	18.5	2.25E+08	1364900
D3YVN7;Q8BFR5;Q8BFR5-2	Gm9755;Tufm	12	5	49.538	452	35.4	13.3	15885000	1323500
Q8VDN2;D3YYN7;Q8VCE0;Q6PIC6	Atp1a1	19	4	112.98	1023	25.5	5.5	10587000	1300400
Q9D2G2;Q9D2G2-2	Dlst	9	2	48.994	454	25.6	4.4	13607000	1252400
P70704;F6WYQ5	Atp8a1	39	4	129.77	1149	41.9	3.3	47017000	1244800
A2A547;P84099	Rpl19	8	2	23.247	194	38.1	8.8	4537000	1235500
Q9CY27;G3UWE1;Q52L67	Tecr	9	2	36.09	308	23.7	6.2	14086000	1228900
P47738;D3YYF3	Aldh2	27	4	56.537	519	62	6	63466000	1216800
Q91VD9	Ndufs1	22	5	79.776	727	43.1	6.5	9710800	1163900
F8WH58	Cntn4	0	1	79.521	714	0	1.5	0	1140100
Q99JY0;D3YXU1	Hadhb	20	5	51.386	475	53.5	9.1	38553000	1131300
P28843	Dpp4	21	3	87.436	760	37.1	3.8	12299000	1120600
Q922Q1;F6UB20;F7DFG6;E0CZH6		12	2	38.194	338	41.1	4.4	9067400	1106200
Q9D8E6	Rpl4	12	3	47.153	419	33.9	6.4	15620000	1075700

Q9D051	Pdhb	9	3	38.937	359	43.2	10.6	8730400	1022200
E9QPU1;Q8CIZ8	Vwf	14	8	309.47	2816	8.2	3.3	6341000	989080
Q63880;Q63880-2	Ces3a	14	3	63.317	571	34.9	7	22733000	962590
Q9CQF9;F7CIP8;D3Z275	Pcyox1	23	4	56.494	505	72.5	11.5	26513000	960530
P35980;G3UZK4;G3UX28;G3UZJ6;G3UYV6	Rpl18	6	2	21.644	188	36.2	11.2	5571500	932100
P56480	Atp5b	27	5	56.3	529	68.4	13.6	1.2E+08	925050
Q63836;G3UYY2	Selenbp2;Selenk	13	2	52.609	472	46	4.2	5432300	877460
Q8K2B3	Sdha	18	5	72.585	664	29.5	6.8	12974000	876060
P14148;F6XI62	Rpl7	19	4	31.419	270	56.7	14.4	11265000	862490
D3Z041;P41216;D3Z457;F6WNZ2;D3YVF6	Acsl1	33	2	78.034	699	50.9	3.1	40095000	857160
P02088;A8DUK4;E9Q223	Hbb-b1;Hbbt1	8	2	15.84	147	59.9	17	36113000	845360
Q6DFW5;E9Q0M7;F6TYE6	Atp11b	38	5	133.53	1175	38	4.9	1801100	843000
E9Q557;E9PZW0	Dsp	32	6	332.91	2883	12.7	2.3	11971000	830060
A2A839;A2A838;A2A842;A2A841	Epb4.1;Epb41	7	5	72.423	639	15.2	11.7	1695300	828330
E9Q9J0;E9Q4P0;E9Q5F6;E9QNP0	Uba52;Kxd1;Ubc	7	1	10.914	96	57.3	13.5	17683000	798640
Q9QXZ6;E9Q0W2;Q91YY5	Slco1a1	12	4	74.396	670	20.7	4.8	3425300	795980
A2ATU0	Dhtkd1	13	5	102.79	921	23.2	5.9	6048100	784070
B0V2N8;B0V2N7;P07356;B0V2N5	Anxa2	9	2	19.596	176	55.7	11.9	3604100	773210
Q60759	Gcdh	15	1	48.605	438	58	3.2	16586000	771330
K9J7B2;E9PXN7;D3Z748;	Ugt1a6b	19	3	60.49	531	44.8	5.8	9798700	741240
P16858;F8WJL5;M0QWZ0	Gapdh;Gm7293	8	2	35.81	333	31.2	4.8	4031300	734090
P14869;E9Q070;D3YVM5	Rplp0;Gm8730	13	1	34.216	317	49.5	3.5	16688000	727790
Q8K154;E9PZ30;D3YUX6;Q80X89;Q6PDD0	Ugt2b34	17	1	60.876	532	50.8	2.3	14692000	675360
Q8VCT4;E9PYP1;D3Z5G7;P23953	Ces1d	17	1	61.787	565	46.2	1.6	20286000	627290
P62918	Rpl8	10	2	28.024	257	44	9.7	6992300	625520
O08749	Dld	15	4	54.272	509	46.4	10.2	13011000	615000
E9PUX4;P47911	Gm5428;Rpl6	8	2	33.59	296	27	10.1	8053600	607200
P50427	Sts	3	2	66.59	624	6.6	3.4	658350	599260
B1AR28;P50544	Acadvl	34	1	68.55	634	61.8	2.1	30514000	592230
Q9CZ13	Uqcrc1	15	2	52.851	480	46.2	4.6	20133000	558650
Q60597-2;Q60597	Ogdh	29	4	115.42	1013	37.4	4.4	17258000	547920
P20918	Plg	3	6	90.807	812	6.3	7.9	1178500	523370
Q3USB7;Q8K394	Plcl1;Plcl2	0	1	122.67	1096	0	0.8	0	518660
Q8CHT0	Aldh4a1	13	3	61.84	562	30.6	5.9	12534000	505440

P54869;Q8JZK9	Hmgcs2	26	3	56.822	508	68.5	9.4	79446000	503860
Q9JLF6	Tgm1	5	2	89.825	815	7.9	2.6	1405300	487770
L7N202;P12970;D3YXT4	Rpl7a;Gm4978	7	1	29.964	266	27.1	3	5255200	477490
P14246;E9PXR7	Slc2a2	5	1	57.106	523	7.1	2.1	3553900	467580
P33267	Cyp2f2	19	1	55.948	491	51.3	2.4	17490000	464090
Q9CR57	Rpl14	9	1	23.564	217	35.9	6	7772700	457820
P16460;J3QNG0	Ass1;Gm5424	20	3	46.584	412	72.6	7.3	15487000	451180
P21981;G3UXE8	Tgm2	10	2	77.06	686	21.9	3.6	4398700	426450
Q6ZWN5;F7CJS8;D3YWH9	Rps9	9	2	22.591	194	47.4	9.8	4832600	422890
Q3UZ09;Q8CG16	C1rl	8	2	53.435	482	21.4	3.9	5258200	419950
Q9DB20;F7D3P8;F6XVM5	Atp5o	14	1	23.363	213	66.7	3.8	41099000	413310
Q99LB2	Dhrs4	14	1	29.884	279	55.6	4.7	16790000	404410
P61620;Q9JLR1;A2ATT9	Sec61a1	10	2	52.264	476	25.6	4.4	5191800	400850
B1AU25;Q9Z0X1	Aifm1	22	3	66.113	608	49.3	4.9	21274000	398120
Q9CQS8;E9PW43	Sec61b	3	1	9.9583	96	35.4	15.6	2718400	398100
P47963;D3YX54	Rpl13	11	1	24.305	211	46.4	5.2	9432500	396070
P62908;D3YV43	Rps3	13	3	26.674	243	65.4	16.5	10047000	394810
E9Q3X0;Q9EQK5;D3Z2N7	Mvp	12	2	96.856	870	24.1	2.5	3627700	387660
Q80XL6;D3YTQ5;F6U473	Acad11	16	3	87.365	779	28.8	3.9	5963100	375490
Q91WU0	Ces1f	15	1	61.612	561	46.5	2.1	9370400	366950
Q3UV17	Krt76	11	4	62.844	594	10.4	6.4	237460	363750
Q91ZA3;H3BL62	Pcca	19	2	79.921	724	38.1	3.2	8114800	359830
P62717;F6YJW4	Rpl18a;Gm1754	6	2	20.732	176	38.6	10.2	3973300	353440
H3BJ51;Q64FW2;F7CW61;H3BKK1	Retsat	4	2	61.168	548	11.1	4	757270	338580
P25444;L7N240;D3Z659	Rps2;Gm5786	10	2	31.231	293	40.6	6.5	9198700	335040
P62702;Q3V1Z5;D3Z2E6	Rps4x;Rps4l	16	1	29.597	263	65.4	2.7	17648000	327950
Q3ULD5	Mccc2	10	1	61.378	563	32.3	1.6	4738800	317380
Q9DCP2	Slc38a3	3	2	55.591	505	9.3	4.8	1834900	310510
Q62425	Ndufa4	7	1	9.3267	82	90.2	9.8	23788000	305460
P16015	Ca3	6	1	29.366	260	36.2	4.6	1973600	301800
P27773;F6Q404	Pdia3	12	1	56.678	505	28.5	2.6	13103000	298470
D3Z6C3;P97351;D3Z7W7	Rps3a	14	1	29.824	264	40.2	4.9	10253000	290990
P61255;B1ARA5;B1ARA3;K4DI68	Rpl26	7	3	17.258	145	34.5	18.6	7106800	289440
P67778;Q5SQG5	Phb	15	2	29.82	272	67.6	7.7	32553000	287070

P07724	Alb	14	4	68.692	608	32.4	9.4	8804200	281710
Q62261;Q62261-2	Sptbn1	26	2	274.22	2363	17.5	1.2	4999200	278450
Q61694;Q61767;E9Q007	Hsd3b5	14	1	41.892	373	63.3	2.9	12732000	277930
P57780;E9Q2W9;D3Z0L8;D3Z761	Actn4	11	2	104.98	912	22	2.7	2896500	273060
Q7JCA9;P00416	COX3;mt-Co3	1	1	29.936	261	5.4	5.4	754870	270180
O54749;Q924D1;G3UXT0	Cyp2j5	22	2	57.783	501	63.9	5	8447900	268050
P62281	Rps11	15	2	18.431	158	70.9	10.1	8250800	266640
Q8BGH2	Samm50	14	3	51.863	469	35.4	8.3	5219200	264560
P46978;D3YZN5	Stt3a	2	1	80.597	705	3	1.4	988110	257260
P32020;P32020-2;A2APS4	Scp2	20	1	59.125	547	39.9	2.2	22594000	253380
P11725;Q8R1A8	Otc	26	3	39.764	354	82.2	13.8	88435000	249880
Q3U3J1;P50136	Bckdha	10	2	50.773	446	30.9	7.4	7119400	249320
Q91W43	Gldc	14	1	113.27	1025	20.3	1.6	5590700	248870
Q9CQA3	Sdhb	8	1	31.814	282	32.6	3.9	9204700	245760
Q8JZU2;F6VVY4	Slc25a1	17	1	33.931	311	60.1	2.6	12139000	244670
P50285;Q3UNX7;D3Z0T2;Q8C9C1	Fmo1	14	2	59.914	532	36.5	4.5	7012100	242340
Q78PY7;Q3TJ56;E9Q3E9	Snd1	20	2	102.09	910	28.6	2.5	3487300	236680
Q9QYG0;Q9QYG0-2	Ndrg2	11	2	40.789	371	55.5	6.7	8467700	233080
P61027;Q3TYH2;Q9DD03;Q8K386	Rab10	3	1	22.541	200	16.5	6	406850	231210
Q3V1K7;Q8BXB6	Slco2b1	1	1	75.581	693	1.7	1.9	202370	223930
Q9EPF6;K7N641	Olfr704;Olfr694	1	1	35.12	315	2.2	2.2	119050	222980
J3KMG3;Q8BI84	Mia3	4	1	174.31	1565	4.7	1.8	774910	221850
G3XA10;Q8VEK3	Gm28062;Hnrnp	4	1	86.805	793	7.7	1	865940	219270
A2AQR0;Q64521	Gpd2	15	2	82.829	745	28.1	2.7	4228100	218440
Q9D3D9	Atp5d	4	1	17.6	168	41.1	8.3	5486400	216690
P16406;F7B9G4;F6YHW2	Enpep	7	1	107.96	945	8.7	1.4	663970	213440
Q8JZQ2	Afg3l2	6	1	89.518	802	15.2	1	2108300	212810
Q00896;P07758	Serpina1c	8	1	45.823	412	32	2.4	3676600	202500
Q9CQN1	Trap1	14	3	80.208	706	29.6	5.2	3904500	202170
Q8K2X1-2;Q8K2X1	Atp10d	10	2	156.34	1400	12.1	1.9	1801100	201200
O88455	Dhcr7	5	1	53.918	471	10.8	1.7	1076600	197890
Q921L6;Q60598	Cttn	5	2	57.086	509	12.6	3.3	673020	195800
P15105;D3YVK1;D3Z121	Glul	3	1	42.119	373	6.7	2.7	1066300	194180
Q569X9;E9Q7C	Cyp2c67;Cyp2c4	15	1	56.144	491	35.6	2.9	5943400	190670

Q9Z2I0	Letm1	9	1	82.988	738	19.1	1.6	2250500	187980
Q68FG2	Sptbn2	9	3	270.92	2388	6.3	1.5	717780	178750
G5E8U1;P58735;D3Z4W4	Slc26a1	6	1	77.696	720	12.5	1.7	1130000	177270
Q6PF96;Q921G7	Etfdh	17	3	61.16	556	43.2	6.7	14137000	175460
P55258;P61028	Rab8a;Rab8b	4	1	23.668	207	20.3	5.8	425410	174690
E9Q1Y3;E9Q414;E9Q4G4	Apob	8	3	503.9	4456	3.3	0.6	3395000	173710
P19783;M0QWX7	Cox4i1	11	1	19.53	169	68	5.9	19206000	172580
Q9QXY6;Q9WVK4;Q9EQP2	Ehd3	11	2	60.82	535	32.5	3.4	3168600	169010
Q8VDJ3	Hdlbp	13	1	141.74	1268	14.2	0.9	3101600	167450
Q8BU14	Sec62	0	1	45.58	398	0	3	0	162410
Q80UM7	Mogs	10	1	91.83	834	16.3	1.6	2841400	160520
Q05421	Cyp2e1	22	2	56.804	493	45.6	5.1	14557000	160390
Q9CZW5	Tomm70a	8	1	67.589	611	15.2	2.5	1326900	159830
B1AR35;P24721;J3QMY0	Asgr2	2	1	25.967	226	20.8	3.1	476350	159800
Q99K67;F6VMP2	Aass	21	1	102.97	926	39.5	1	14205000	156580
P11276	Fn1	16	2	272.53	2477	9.8	1.3	5875000	155870
Q8BMF4	Dlat	12	1	67.941	642	33.2	2.3	10858000	152910
Q91YQ5	Rpn1	16	1	68.527	608	37.8	2.8	10754000	151050
P14211	Calr	13	2	47.994	416	32	6.7	13129000	148590
Q9WU79;F6YFQ5;F6PYI8	Prodh	7	1	68.035	599	20.7	1.8	2138900	147420
Q8C2Q8;Q91VR2;A2AKU9	Atp5c1	13	2	30.255	274	53.6	8	22321000	146600
O88569-3;O88569-2;O88569	Hnrnpa2b1	2	1	32.46	301	7.3	4	254970	145760
Q9R099;F8WI46;D3YZH8	Tbl2	8	2	49.583	442	25.8	5.2	4142200	144690
H3BK44;E9PY90	Ctage5	5	1	85.621	761	8.8	1.8	977910	144160
Q9D379;E9PWK1;F6YTS6;D3Z4M3	Ephx1	19	2	52.576	455	56.5	4.8	11997000	140770
P09103;E9Q8G8	P4hb	26	2	57.058	509	54.8	3.7	29411000	136940
P01872;P01873	Ighm	1	1	49.971	454	4.6	2.2	251900	136820
Q9QXX4	Slc25a13	27	2	74.466	676	50.7	2.5	19749000	136490
P29341;F6ZAX1;Q91YZ8	Pabpc1	6	1	70.67	636	11.8	1.7	2791000	136260
P97807-2;P97807;H3BKG7	Fh	13	1	50.052	467	49.7	1.5	10198000	129520
G5E8J2;B7ZW98;E9QNT8	Ank1	14	1	202.52	1848	13.4	0.5	2698000	127800
P68372;J3QNR5;Q9D6F9	Tubb4b	11	1	49.83	445	37.1	2.7	11571000	127060
P00186;P00184	Cyp1a2	19	1	58.183	513	46.4	3.1	13304000	125350
A2AVJ7;Q99PL5;Q99PL5-12	Rrbp1	4	1	158.39	1464	3.3	0.8	940720	121590

Q99LB7;A2AH52;A2AH53	Sardh	23	5	101.68	919	44.6	6.6	16240000	118260
P05202	Got2	22	1	47.411	430	56	1.9	27529000	117940
Q01853	Vcp	6	1	89.321	806	11.4	1	1251900	114860
Q91W64	Cyp2c70	12	1	56.019	489	37.4	2.2	6252400	114460
Q9R1S8	Capn7	0	1	92.563	813	0	1.1	0	113450
Q60936;F7B1B6;Q60936-2	Adck3	15	2	71.742	645	32.2	3.6	8438900	113220
D3YUM1;Q91YT0;D3Z1U9	Ndufv1	15	1	49.913	455	40.4	2	4384600	112130
E9Q035;Q921I1;P47758	Gm20425;Tf	20	1	107.8	978	26.8	1.8	10298000	111440
P62754	Rps6	10	1	28.68	249	39.8	2.8	8208000	108080
E9PYL9;Q9CXW4;D3Z3K1	Gm10036;Rpl11	9	1	20.266	178	58.4	4.5	9437500	106510
F8VQM0;P24822	Akp3;Iap	1	1	60.291	559	1.6	1.6	68470	102760
Q99JB2;A2AG39;F6WI02;A2AG41	Stoml2	4	1	38.384	353	24.1	3.1	1745000	97530
Q9QYC7	Ggcx	3	1	87.194	757	4.4	1.3	482010	96581
F8WJ71;D3Z549;D3Z7I6	Numb	0	1	9.0012	76	0	14.5	0	94088
Q9WV55	Vapa	5	1	27.855	249	39.4	6.4	2845400	92992
E9Q4M2;P54310;Q8CDI9	Lipe	1	1	117.28	1072	1.1	0.9	17058000	91746
B7FAV1;B7FAU9;Q8BTM8	Flna	2	1	274.63	2583	1.1	0.4	2310900	89458
Q9D0F3	Lman1	5	1	57.788	517	21.7	1.5	1064700	89070
E9QAH1;E9PVZ8	Golgb1	5	1	365.29	3197	2.3	0.5	580660	85868
Q9CR67;E0CXY5;Q9CZM3	Tmem33	3	1	28.031	247	11.3	4.9	418680	83599
A2BGG7;P62960	Ybx1	1	1	24.74	214	11.2	10.7	480200	80498
Q31125	Slc39a7	0	1	50.656	476	0	3.2	0	80329
D3Z1D6;D3YWP3	Rpl23a	6	1	17.725	156	39.1	6.4	10356000	78390
Q8VCB3	Gys2	6	1	80.87	704	15.5	2	1056700	75041
Q91X75;P20852	Cyp2a5;Cyp2a4	11	1	56.712	494	33.8	2.2	3103600	74846
P41105	Rpl28	5	1	15.733	137	31.4	8	2241000	74700
O88962	Cyp8b1	6	2	57.706	500	22.2	4.4	1842800	73102
F6SMH4;O88908	Soat2	2	2	31.989	284	9.9	8.5	216980	72964
Q9CXW2	Mrps22	2	1	41.192	359	8.9	3.1	650340	63547
B1B0C7;E9PZ16;Q05793	Hspg2	6	1	469.02	4375	2.3	0.3	943060	63185
Q9EQ20	Aldh6a1	27	2	57.915	535	72.1	3.9	28886000	60454
Q61696;P17879	Hspa1a;Hspa1b	4	2	70.078	641	13.1	3.6	179460	60450
Q9WVD5	Slc25a15	15	1	32.823	301	77.7	5.3	6789500	58899
Q9Z0R9	Fads2	3	1	52.387	444	9.9	2.5	1407700	55451

Q9EP89	Lactb	7	1	60.705	551	24.9	1.6	3136000	49542
A2A5N1;Q9CQV8-2;Q9CQV8	Ywhab	2	1	18.348	159	17.6	8.8	203530	48617
P17426-2;P17426;F6TPX8	Ap2a1	9	2	105.48	955	14.9	2.1	787640	44896
Q99LC5	Etfa	17	1	35.009	333	67	2.1	21945000	44578
P14576;E9PXC0;P14576-2	Srp54;Srp54c	5	1	55.72	504	16.9	2.6	494960	41330
Q91VR5	Ddx1	7	1	82.499	740	13.6	1.8	1225300	40312
Q9EP69	Sacm1l	6	1	66.943	587	13.5	1.2	909040	36182
Q9JKF7	Mrpl39	2	1	38.549	336	10.1	5.7	289970	34507
P50247;A2ALT5	Ahcy	3	1	47.688	432	10.6	2.8	452110	26770
Q5XG73-3;E9QNH7	Acbd5	7	1	52.468	473	17.8	2.3	3336400	24450
P30115	Gsta3	5	1	25.36	221	37.1	8.1	1069400	23117
Q8QZT1	Acat1	15	1	44.816	424	58.5	1.9	28164000	22728
Q99KI0	Aco2	20	2	85.462	780	38.6	2.6	14018000	16069
D3Z1M3;Q6ZQI3	Mlec	2	1	32.399	292	13.7	7.9	241140	13787
Q9CQR2	Rps21	6	1	9.1413	83	65.1	19.3	4379600	13202
Q8VCU1;G5E8K9	Ces3b	11	2	63.006	568	26.9	4.8	3046800	11984
O35114	Scarb2	3	1	54.043	478	11.5	5	382670	11838
P23927	Cryab	4	1	20.069	175	26.3	5.7	1304300	11814
Q3U367;Q9JLJ2	Aldh9a1	6	1	55.887	518	17.6	2.3	1123000	11401
Q9Z2I8;Q9Z2I8-2	Suclg2	16	2	46.839	433	45	5.3	7910900	11276
Q91VA7	Idh3b	2	1	42.194	384	6.5	4.4	622070	10031
O54734	Ddost	7	2	49.027	441	29.3	4.3	6558400	10026
Q9WTP7;F6RP11	Ak3	5	1	25.426	227	30	4.4	4619800	9940.9
Q9DBF1-2;Q9DBF1	Aldh7a1	17	1	55.644	511	48.3	3.3	8484800	9763.9
P61514	Rpl37a	1	1	10.275	92	13	13	319450	9329.6
Q9DBT9	Dmgdh	37	2	97.254	869	56.3	3.1	28931000	9156.4
Q9ERS2	Ndufa13	5	1	16.859	144	32.6	7.6	3101200	9015.6
Q9CPQ1;D3Z6E1	Cox6c	7	1	8.4689	76	82.9	23.7	10587000	7795.8
P28665;P28666	Mug1	21	1	165.3	1476	23.2	1.1	6050700	7685.2
Q78IK2	Usmg5	2	1	6.3814	58	60.3	27.6	6534700	7584.5
D6REF7;Q8R0X7;D3YZT4;D3YY13;D3Z1Z3	Sgpl1	10	1	54.769	487	31	2.7	1878900	7422.2
O08914	Faah	8	1	63.221	579	21.8	2.8	3097900	6970.8
Q8BVA5-2;Q8BVA5		1	1	26.795	236	7.6	7.6	137920	6937.2
Q9JHI5	Ivd	9	1	46.325	424	35.4	2.4	6266100	6879.1

J3QJX3;Q9Z2G6-2;Q9Z2G6	Sel1l	3	1	82.425	740	5	0.9	323920	6789.4
G3UY29;E9Q3P9;F8WGS1	Rab11b;Rab11a	3	1	16.924	149	19.5	7.4	853510	6736
O35857;F7C189	Timm44	5	1	51.091	452	15	2.7	1610800	6682.6
Q8BH95;F6T930	Echs1	11	1	31.474	290	63.8	4.5	9357300	6009.6
P51658	Hsd17b2	9	1	41.835	381	37.3	6	3892100	5945.5
Q99L13	Hibadh	10	1	35.44	335	54.6	4.8	3448000	5880.1
B8JK33;B8JK32;Q9D0E1-2;Q9D0E1	Hnrnpm	1	1	68.161	640	1.7	1.7	99929	5863.2
Q9CQ54	Ndufc2	4	1	14.164	120	32.5	7.5	2251800	5741.7
P07759;Q03734;G3X8T9	Serpina3k	11	1	46.879	418	38.3	2.4	6974600	5721.4
P56593;B2RXZ2	Cyp2a12	17	1	56.179	492	44.5	2.4	6916300	5607.3
Q8K169;Q3UWB9;P17717	Ugt2b5;Ugt2b17	23	1	60.873	530	64	2.6	41068000	5591.1
P14115	Rpl27a	7	1	16.605	148	39.9	8.8	3535900	5485.5
P23116	Eif3a	3	1	161.93	1344	2.3	0.6	160790	5478.3
D3Z600;O09012-2;O09012	Pex5	1	1	69.836	632	2.4	2.4	81852	5348.1
Q9WVL0	Gstz1	4	1	24.275	216	24.5	5.1	1522400	5321
Q3TLP5;F6YTG0	Echdc2	4	1	31.852	296	21.6	5.7	1785600	5310.1
Q9WV68	Decr2	7	1	31.3	292	28.1	4.1	2647300	5307.8
P56379	Mp68	2	1	6.6979	58	63.8	13.8	1505700	5248.1
P14131	Rps16	10	1	16.445	146	52.7	7.5	9367700	5244.3
Q9CWD8;F7D3P0	Nubpl	2	1	34.139	319	9.4	3.1	316080	5198.7
Q64458;Q3UT49;H3BLM0	Cyp2c29	28	1	55.715	490	72.2	2.4	34285000	5165.8
Q9CQ75	Ndufa2	5	1	10.916	99	56.6	14.1	2964700	5027.3
Q9CZX8;D3Z722	Rps19	10	2	16.085	145	60	13.8	11025000	4937.8
Q9DCM0	Ethe1	5	1	27.738	254	40.2	11.4	1086000	4933
P17665;J3QNB4;B8JJA9	Cox7c	4	1	7.3325	63	65.1	14.3	3217000	4883.6
P27046;F6QMB7	Man2a1	20	1	131.63	1150	23.4	0.9	5494700	4832.6
P29758	Oat	8	1	48.354	439	26	4.1	3310900	4826
P62270;F5H8M6	Rps18;Gm1026(	8	1	17.718	152	52.6	7.9	6476800	4743.2
Q9CQH3;D3Z568	Ndufb5	6	1	21.71	189	31.2	5.8	3493600	4734
Q9CVB6;D3YXG6	Arpc2	3	1	34.357	300	12.7	3	509950	4523.8
Q9DBG3;Q9DBG3-2	Ap2b1	12	1	104.58	937	16.9	1.8	3733600	4504.8
D3Z5K6;Q9CQV7	Dnajc19	2	1	12.191	110	29.1	16.4	292060	4385
F7ARZ1;A7M7Q8	Mrps23	2	1	13.295	120	17.5	9.2	437460	4304
D3YW52;Q61838	Pzp;A2m	8	1	167.28	1507	9.1	0.8	1532800	4279

Q07417;F6RAZ3	Acads	11	1	44.889	412	35	2.4	15409000	4217.1
P62204;Q3UKW2;Q9D6P8	Calm1	3	1	16.837	149	38.3	8.1	2071900	4208.3
Q8CHQ9	Cml2	9	1	26.424	238	57.1	4.2	8754400	4158.1
P45952;D6RFD7	Acadm	13	1	46.481	421	43	4.5	10271000	4129.2
Q9D1I6	Mrpl14	2	1	15.874	145	18.6	6.9	1010300	4127.5
Q9CQE1;B1AWZ5	Nipsnap3b	6	1	28.308	247	50.6	5.3	1542200	4107.4
H3BJG4;E9Q512;Q8BVJ9	Trip11	9	1	194.66	1691	6.2	0.5	1606300	4029.3
Q9WU19	Hao1	7	1	41.001	370	23.2	2.4	797700	3859.9
E9PZ00;Q8BFQ1	Psap	5	1	60.672	551	11.4	2.7	878640	3808.7
O88451;D6RFQ4;Q6PHA1	Rdh7	10	1	35.66	316	43	3.2	13513000	3785.5
D3YYT0;P15116	Cdh2	3	1	93.856	849	4.5	1.1	661930	3709.2
Q9R0H0-2;Q9R0H0;A2A848	Acox1	10	1	74.717	661	21.8	3.6	2944800	3655.1
P51410;G3UW34	Rpl9	10	1	21.881	192	71.9	11.5	9755900	3602
P85094	Isoc2a	7	1	22.417	206	50	7.8	4841800	3542.4
P46656	Fdx1	2	1	20.123	188	13.8	4.8	814240	3535.9
Q8BWF0	Aldh5a1	5	1	55.968	523	12.4	3.4	866870	3301.2
Q3U1J4	Ddb1	4	1	126.85	1140	5.1	0.9	311190	3128.3
Q8R0Y6	Aldh1l1	4	1	98.708	902	9.1	2.4	876230	2926.7
Q99K47;E9PV24	Fga	8	1	61.325	557	15.1	2.9	2129700	2487.5
D6RHS6;D3Z1V4;P70296	Pebp1	1	1	14.835	136	13.2	13.2	45790	1010.2
Q8JZR0	Acsl5	14	2	76.205	683	29.9	2.6	4945600	0
P27659;Q9CQ09;E9PWZ3	Rpl3	12	2	46.109	403	40.7	4	20273000	0
P02089	Hbb-b2	9	2	15.878	147	80.3	17	5672600	0
Q62452	Ugt1a9	9	2	60.007	528	18.8	4.2	263790	0
Q921X9	Pdia5	5	2	59.266	517	16.6	3.9	2238800	0
Q8VCC2	Ces1	5	2	62.679	565	10.8	3.7	1542300	0
Q64459;Q9JMA7;Q64481	Cyp3a11	25	1	57.854	504	70.6	1.4	28320000	0
Q3UEP4;D3YUP6;D3YWN0;D3YXD5	Ugt2b36	22	1	61.041	530	57.2	2.6	21716000	0
Q9DBM2	Ehhadh	22	1	78.301	718	48.6	1	7783000	0
Q925I1;Q925I1-2	Atad3;Atad3a	19	1	66.741	591	34.9	1.2	9246700	0
Q9DB77	Uqcrc2	15	1	48.234	453	50.3	2	26892000	0
Q6XVG2	Cyp2c54	15	1	55.857	490	25.5	2.4	4399800	0
Q91X77;Q91X77-2	Cyp2c50	13	1	55.764	490	23.9	2.4	871600	0
Q61941;Q8C9V5	Nnt	12	1	113.84	1086	13.7	0.6	5885500	0

P24456	Cyp2d10	12	1	57.233	504	35.5	1.4	5761400	0
Q8BWQ1	Ugt2a3	10	1	61.119	534	35.4	2.2	6033100	0
Q9CW42;D3YZZ3		10	1	37.978	340	45.6	2.4	4449600	0
P37040;Q05DV1	Por	10	1	77.043	678	23.7	1.2	3323000	0
P99024	Tubb5	10	1	49.67	444	33.1	2.7	1074700	0
P11499;D3Z1R1;E9PX27	Hsp90ab1	8	1	83.28	724	13.5	1.7	745450	0
A1BN54;Q7TPR4;Q9JI91;O88990	Actn1	8	1	102.72	887	14.4	1.7	676300	0
Q7TMM9	Tubb2a	8	1	49.906	445	24.9	2.7	119140	0
P60867	Rps20	7	1	13.373	119	47.9	9.2	16849000	0
P10126;D3YZ68;D3Z3I8;P62631	Eef1a1;Eef1a2	7	1	50.113	462	28.8	1.5	4563200	0
B1AXW5;B1AXW6	Prdx1	7	1	18.927	170	35.9	4.7	2030600	0
P19253;E9Q5A0;D3YY61	Rpl13a	5	1	23.464	203	31.5	5.4	2461500	0
Q8CC88;Q8CC88-2	Vwa8	5	1	213.42	1905	3.6	0.4	1074100	0
E9PZB3	Gm5093	5	1	19.876	174	39.1	4.6	121890	0
P98200	Atp8a2	4	1	129.42	1148	4.2	1	1268200	0
P62911;A2AD25;P17932	Rpl32	3	1	15.86	135	22.2	5.2	2277300	0

Table S4 Abundant Proteins in Mouse Testes

Majority protein IDs	Protein names	Gene names	Peptides Testis	Mol. weight [kDa]	Sequence length	Sequence coverage Testis [%]	Intensity Testis
Q6UQ17	Phospholipid-transporting ATPase IK	Atp8b3	55	151.95	1335	42.2	45604000
E9QKK8;Q9QZW(	Phospholipid-transporting ATPase;Pho:	Atp11c	52	127.81	1116	39.2	58310000
Q6DFW5;E9Q0M	Phospholipid-transporting ATPase	Atp11b	48	133.53	1175	43.5	60457000
P20029	78 kDa glucose-regulated protein	Hspa5	41	72.421	655	56.2	25040000
P70704;F6WYQ5	Phospholipid-transporting ATPase IA;P	Atp8a1	38	129.77	1149	39.6	17741000
E9Q3G7;P98197	Phospholipid-transporting ATPase;Prol	Atp11a	37	130.68	1142	33.8	51481000
P98200	Phospholipid-transporting ATPase IB	Atp8a2	37	129.42	1148	27.5	13082000
Q61301;E0CXB9;:	Catenin alpha-2	Ctnna2	31	105.28	953	41.4	11786000
Q8CAQ8-2;Q8CA(	MICOS complex subunit Mic60	Immt	26	82.928	746	46.8	3602000
P17156	Heat shock-related 70 kDa protein 2	Hspa2	24	69.641	633	34	6219800
Q8BMS1	Trifunctional enzyme subunit alpha, mi	Hadha	23	82.669	763	36.3	3974200
Q8K2X1-2;Q8K2X	Probable phospholipid-transporting AT	Atp10d	21	156.34	1400	18	3174300
Q02248;E9Q6A9	Catenin beta-1	Ctnnb1	21	85.47	781	33.7	7689400
Q03265;D3Z6F5	ATP synthase subunit alpha, mitochon(	Atp5a1	21	59.752	553	47	3550700
Q8VEK0;D3YVV1	Cell cycle control protein 50A	Tmem30a	19	41.06	364	49.7	90230000
Q8R127	Saccharopine dehydrogenase-like oxid(	Sccpdh	19	47.129	429	35.4	11803000
P14211	Calreticulin	Calr	19	47.994	416	42.5	6463600
P63017;Q504P4	Heat shock cognate 71 kDa protein	Hspa8	18	70.87	646	29.3	1292400
P08113;F7C312	Endoplasmin	Hsp90b1	18	92.475	802	24.9	2933000
P38647	Stress-70 protein, mitochondrial	Hspa9	17	73.46	679	36.1	2512200
O54827	Probable phospholipid-transporting AT	Atp10a	17	168.79	1508	15.1	2352600
P63260;P60710;E	Actin, cytoplasmic 2;Actin, cytoplasmic	Actg1;Actb	16	41.792	375	51.7	5040200
P67778;Q5SQG5	Prohibitin	Phb	15	29.82	272	57.7	4844700
P35564	Calnexin	Canx	14	67.277	591	31.3	5468400
P68372;J3QNR5;(	Tubulin beta-4B chain;Tubulin beta-4A	Tubb4b;Tul	14	49.83	445	44.9	2922800
P52194	Calmegin	Clgn	13	69.43	611	34	8341800
P35487	Pyruvate dehydrogenase E1 componer	Pdha2	13	43.412	391	32.7	2376800
D3YYT0;P15116	Cadherin-2	Cdh2	12	93.856	849	19	5221700
O35129	Prohibitin-2	Phb2	12	33.296	299	48.5	3142000
P27786	Steroid 17-alpha-hydroxylase/17,20 lya	Cyp17a1	12	57.637	507	34.1	1523200

P68134;P68033;I	Actin, alpha skeletal muscle;Actin, alph	Acta1;Actc1	11	42.051	377	26.8	316560
P56480	ATP synthase subunit beta, mitochond	Atp5b	11	56.3	529	34.6	2401900
Q99JY0	Trifunctional enzyme subunit beta, mit	Hadhb	11	51.386	475	25.3	2698000
P99024	Tubulin beta-5 chain	Tubb5	11	49.67	444	33.8	540750
P10126;D3Z3I8;D	Elongation factor 1-alpha 1;Elongation	Eef1a1;Eef1	10	50.113	462	27.1	2447000
P02089	Hemoglobin subunit beta-2	Hbb-b2	10	15.878	147	67.3	981460
J3QPE8;J3QMG3;	Voltage-dependent anion-selective cha	Vdac3	10	30.695	283	37.5	1985400
G5E902;Q8VEM8	Phosphate carrier protein, mitochondr	Slc25a3	10	39.736	358	26.3	1657800
Q9Z2I9	Succinyl-CoA ligase [ADP-forming] subu	Sucla2	10	50.113	463	23.3	1289500
P63038;P63038-2	60 kDa heat shock protein, mitochondr	Hspd1	10	60.955	573	27.9	746080
P02088;A8DUK4;	Hemoglobin subunit beta-1	Hbb-b1;Hb	9	15.84	147	68.7	8390600
P20152	Vimentin	Vim	9	53.687	466	21	418150
Q5SXR6;Q68FD5	Clathrin heavy chain;Clathrin heavy cha	Cltc	9	191.98	1679	6.7	653340
Q76LV0;O70325-	Glutathione peroxidase;Phospholipid h	Gpx4	9	29.193	253	38.3	3820200
P68373;P68369;I	Tubulin alpha-1C chain;Tubulin alpha-1	Tuba1c;Tub	9	49.909	449	29.4	3424000
G3UX26;Q60930;	Voltage-dependent anion-selective cha	Vdac2	9	30.446	283	46.6	3083700
Q8BMF4	Dihydrolipoyllysine-residue acetyltrans	Dlat	9	67.941	642	20.2	2335500
Q8QZT1	Acetyl-CoA acetyltransferase, mitochoi	Acat1	9	44.816	424	36.6	1191000
Q8BMK4	Cytoskeleton-associated protein 4	Ckap4	9	63.691	575	25.6	808520
O08749	Dihydrolipoyl dehydrogenase, mitocho	Dld	9	54.272	509	21.8	711540
P51881	ADP/ATP translocase 2;ADP/ATP transl	Slc25a5	8	32.931	298	26.5	973010
Q9D051	Pyruvate dehydrogenase E1 componer	Pdhb	8	38.937	359	25.1	4451100
Q99KV1	DnaJ homolog subfamily B member 11	Dnajb11	8	40.555	358	27.1	2434600
Q9QZ82	Cholesterol side-chain cleavage enzym	Cyp11a1	8	60.314	526	16.3	1394300
J3KMM5;O55143	Calcium-transporting ATPase;Sarcoplas	Atp2a2	8	109.79	998	12.7	861950
E9PXC0;P14576;I	Signal recognition particle 54 kDa prot	Srp54c;Srp5	8	55.815	504	21	814490
P27773	Protein disulfide-isomerase A3	Pdia3	8	56.678	505	21.8	711390
P05214	Tubulin alpha-3 chain	Tuba3a	8	49.959	450	26.2	91919
Q9DB20;F7D3P8	ATP synthase subunit O, mitochondrial	Atp5o	7	23.363	213	32.9	1003000
P00342;D3YVR7;	L-lactate dehydrogenase C chain;L-lact	Ldhc	7	35.911	332	26.5	747250
Q8C635		Gykl1	7	59.916	549	14.8	725720
Q60597-2;Q6059	2-oxoglutarate dehydrogenase, mitoch	Ogdh	7	115.42	1013	8.5	541380
Q9D8E6	60S ribosomal protein L4	Rpl4	7	47.153	419	18.6	381380
P26231	Catenin alpha-1	Ctnna1	7	100.11	906	8.6	108010

Q3UV17	Keratin, type II cytoskeletal 2 oral	Krt76	6	62.844	594	6.1	0
D3YVN7;Q8BFR5;	Elongation factor Tu;Elongation factor	Gm9755;Tu	6	49.538	452	17	872150
P50427	Steryl-sulfatase	Sts	6	66.59	624	15.4	643320
Q8BFZ9	Erlin-2	Erlin2	6	37.872	340	24.7	633210
Q9QZL9	Dickkopf-like protein 1	Dkkl1	6	26.639	230	26.1	532220
P29341	Polyadenylate-binding protein 1	Pabpc1	6	70.67	636	11.3	505420
Q9WTQ5-2;Q9W	A-kinase anchor protein 12	Akap12	6	169.74	1579	5.8	253300
Q91VD9	NADH-ubiquinone oxidoreductase 75 k	Ndufs1	6	79.776	727	9.5	227070
Q9ERD7	Tubulin beta-3 chain	Tubb3	6	50.418	450	17.8	194870
Q02257	Junction plakoglobin	Jup	5	81.8	745	7.4	338900
Q9JMI7	Testis-expressed sequence 101 protein	Tex101	5	26.998	250	19.6	1456400
Q791V5;A2AFW6	Mitochondrial carrier homolog 2	Mtch2	5	33.499	303	25.1	1003800
P24815;A2AEP8;A	3 beta-hydroxysteroid dehydrogenase/	Hsd3b1	5	42.062	373	19	793060
Q9WUM5	Succinyl-CoA ligase [ADP/GDP-forming]	Suclg1	5	36.154	346	21.7	737440
B2RUF0		Ybx2	5	38.08	359	19.2	714170
Q3V132	ADP/ATP translocase 4;ADP/ATP transl	Slc25a31	5	35.257	320	16.6	564440
Q8K2B3	Succinate dehydrogenase [ubiquinone]	Sdha	5	72.585	664	8.4	553500
Q8K592	Anti-Muellerian hormone type-2 recep	Amhr2	5	61.194	568	13.2	529880
Q9WU65	Glycerol kinase 2	Gk2	5	60.629	554	10.8	508020
P47738	Aldehyde dehydrogenase, mitochondri	Aldh2	5	56.537	519	14.6	498480
Q64435;K9J7B2;I	UDP-glucuronosyltransferase 1-6;UDP-	Ugt1a6;Ugt	5	60.438	531	11.5	479820
Q9CPQ1	Cytochrome c oxidase subunit 6C	Cox6c	5	8.4689	76	52.6	415560
Q9CR57	60S ribosomal protein L14	Rpl14	5	23.564	217	26.7	362420
G3UZK4;P35980	60S ribosomal protein L18	Rpl18	5	15.245	136	33.8	351650
H3BKM0;H3BIY9;	AP complex subunit beta;AP-2 complex	Ap2b1	5	101.36	913	6.4	348800
Q91YQ5	Dolichyl-diphosphooligosaccharide--pr	Rpn1	5	68.527	608	11.2	303150
F8VQ03;Q6P907		Adam3	5	91.443	822	6.3	291780
E9PWK1;Q9D379	Epoxide hydrolase 1	Ephx1	5	50.969	441	11.6	206920
E9QAH1;E9PVZ8		Golgb1	5	365.29	3197	1.8	169690
E9Q7L0		Ogdhl	5	116.6	1029	5.7	62965
P16858;F8WJL5;I	Glyceraldehyde-3-phosphate dehydrog	Gapdh;Gm	4	35.81	333	16.8	290050
P24369	Peptidyl-prolyl cis-trans isomerase B	Ppib	4	23.713	216	19.4	447550
Q91VB8;P01942	Hemoglobin subunit alpha	haemaglob	4	15.112	142	32.4	4138000
P62983;E9Q9J0;E	Ubiquitin-40S ribosomal protein S27a;U	Rps27a;Uba	4	17.951	156	31.4	1076900

O88531;B1B0P8;	Palmitoyl-protein thioesterase 1	Ppt1	4	34.49	306	19.6	1458300
Q810Q5;A2AK37	Normal mucosa of esophagus-specific g	Nmes1;AA4	4	9.5842	83	49.4	1368200
Q8JZU2;F6VVY4	Tricarboxylate transport protein, mitoc	Slc25a1	4	33.931	311	13.8	466550
Q9JIQ3;D3Z3K5;D	Diablo homolog, mitochondrial	Diablo	4	26.82	237	22.8	458150
D3Z1D6;D3YWP3	60S ribosomal protein L23a	Rpl23a	4	17.725	156	32.1	454400
P54869	Hydroxymethylglutaryl-CoA synthase, r	Hmgcs2	4	56.822	508	12	410130
E9QAL4;D3YXQ5;	Phospholipid-transporting ATPase;Pho	Atp8b2	4	134.83	1190	4.6	409270
P35293	Ras-related protein Rab-18	Rab18	4	23.035	206	28.6	383890
P14869	60S acidic ribosomal protein P0	Rplp0	4	34.216	317	13.2	371860
P41105	60S ribosomal protein L28	Rpl28	4	15.733	137	31.4	348910

Table S5 Abundant Proteins from Mouse Kidney

Protein IDs	Protein nar	Gene name	Peptides	Sequence c	Mol. weigh	Sequence l	Intensity
E9Q3G7;F6	Phospholip	Atp11a	58	37.4	130.68	1142	1.15E+09
P98197	Probable p	Atp11a	58	37.1	135.5	1187	884160
F6Q8D3;Q9	Phospholip	Atp11c	45	36.7	129.11	1129	1.93E+08
E9QKK8;Q9	Phospholip	Atp11c	44	36.7	127.81	1116	1037400
F6WYQ5;P7	Phospholip	Atp8a1	33	31.6	131.28	1163	29633000
Q6DFW5;E	Phospholip	Atp11b	30	25.7	133.53	1175	42200000
A2ARV4;A2	Low-densit	Lrp2	27	8.4	519.2	4660	5491100
Q8VEK0;D3	Cell cycle c	Tmem30a	23	47.8	41.06	364	3.79E+08
Q8VDN2;D	Sodium/po	Atp1a1	18	22.6	112.98	1023	7350600
P20029	78 kDa gluc	Hspa5	18	35.1	72.421	655	5093200
Q91WV7	Neutral anc	Slc3a1	15	25.1	78.117	685	8959000
O35488;A2	Very long-c	Slc27a2	15	34.4	70.422	620	6595800
Q03265;D3	ATP syntha	Atp5a1	12	27.8	59.752	553	4220600
P63260;P6	Actin, cytop	Actg1;Actb	10	34.1	41.792	375	9986600
P38647	Stress-70 p	Hspa9	9	18.3	73.46	679	1511900
G5E902;Q8	Phosphate	Slc25a3	8	22.1	39.736	358	4707900
Q7TMS5;D	ATP-bindin	Abcg2	8	15.4	72.977	657	3169000
Q8K2X1-2;	Probable p	Atp10d	8	7.5	156.34	1400	1484600
P68134;P6	Actin, alpha	Acta1;Actc	8	24.4	42.051	377	326650
P28825	Meprin A s	Mep1a	7	13.8	84.23	747	2145900
Q9JIL4;D3Y	Na(+)/H(+)	Pdzk1	7	20.2	56.498	519	1607700
P26040	Ezrin	Ezr	7	13.3	69.406	586	1369500
P63017;Q5	Heat shock	Hspa8	7	14.2	70.87	646	1186500
P51881;P4	ADP/ATP tr	Slc25a5;Slc	6	22.1	32.931	298	3250900
P56480	ATP syntha	Atp5b	6	19.1	56.3	529	2198100
P70441	Na(+)/H(+)	Slc9a3r1	6	25.1	38.6	355	1996300
O55125;Q5	Protein Nip	Nipsnap1	6	22.5	33.363	284	1860300
Q91WU2;C	Solute carr	Slc22a7	6	12.8	59.613	540	1740400
P10126;D3	Elongation	Eef1a1;Eef	6	15.8	50.113	462	1127100
Q61847;Q6	Meprin A s	Mep1b	6	11.6	79.5	704	1077700
P24270;A2	Catalase	Cat	6	15.9	59.795	527	1065800
Q7TSG6;P2	Radixin;Mc	Rdx;Msn	6	15.4	46.367	389	296310

Q7TNG8	Probable D	Ldhd	5	13.8	51.847	484	1530800
Q8K2B3	Succinate c	Sdha	5	7.4	72.585	664	1302900
Q80XN0;D3	D-beta-hyd	Bdh1	5	15.2	38.299	343	1147600
Q9DB20;F6	ATP syntha	Atp5o	5	26.3	23.363	213	912930
P14094	Sodium/po	Atp1b1	4	15.5	35.194	304	2078500
A7E1Z5;E9(	Anion exch	Slc4a4	4	5.7	120.46	1070	771950
P68373;P0.	Tubulin alp	Tuba1c;Tuk	4	14.3	49.909	449	685610
P21981	Protein-glu	Tgm2	4	6.3	77.06	686	562170
Q8BMS1	Trifunction	Hadha	4	6.4	82.669	763	440590
P55096	ATP-bindin	Abcd3	4	7.9	75.474	659	406790
E9Q800;Q8	MICOS con	Immt	4	7.8	75.6	679	304710
E9Q9J0;E9(	Ubiquitin-6	Uba52;Kxd	3	35.4	10.914	96	2196100
Q78IK2	Up-regulat	Usmg5	3	44.8	6.3814	58	1229400
F8WHP8;P!	ATP syntha	Atp5j2	3	31.6	9.0054	76	796320
Q9CQA3	Succinate c	Sdhb	3	11.7	31.814	282	681730
E9Q415;P0	Low affinity	Fcgr2b;Fcgr	3	10.9	35.049	312	620250
P62806	Histone H4	Hist1h4a	3	17.5	11.367	103	607760
Q9QZD8	Mitochond	Slc25a10	3	11.1	31.715	287	555240

Table S6 Control

Protein IDs	Protein names	Gene names	Peptides	Mol. weight [kDa]
Q5SXRG	Clathrin	Cltc	28	193631
D3Z6F5	ATP sunthase subunit alpha	Atp5a1	13	59830
Q8VDN2	Sodium/Potassium-transporting ATPase alpha-1	Atp1a1	11	114221
P17182	Alpha-enolase	Enol	7	47453
P99024	Tubulin beta-5 chain	Tubb5	11	50095
Q6PIC6	Sodium/Potassium-transporting ATPase alpha-3	Atp1a3	14	113045
P52480	Pyruvate kinase isozyme	Pkm2	7	58461
Q62261	Spectrin beta chain, brain 1	Sptbn1	19	274908
P68372	Tubulin beta-4B chain	Tubb4b	7	50255
P15409	Rhodopsin	Rho	5	39614
P05213	Tubulin alpha-1B	Tuba1b	7	50804
Q9Z1R9	MCG124046	Prss1	13	26802
P20612	Guanine nucleotide-binding protein G(t)	Gnat1	7	40397
P63017	Heat Shock Protein cognate 71KDa protein	Hspa8	6	71055
P51881	ADP/ATP translocase 2	Slc25a5	5	33138
P17156	Heat Shock related 70KDa protein 2	Hspa2	5	69884
A3KGU5	Spectrin alpha 2	Spna2	28	283519
A2AQ07	Tubulin beta-1	Tubb1	4	51093
P01868	Ig gamma-1 chain C	Ighg1	6	36252
Q8BFZ9	Erlin-2	Erlin2	3	38077
